# Supplementary material for: Assessing the global implications of the COVID-19 pandemic on the cervical cancer elimination initiative
Source: PLOS Glob Public Health. 2025 Apr 28;5(4):e0004419. doi: 10.1371/journal.pgph.0004419 (PMC12036855; doi:10.1371/journal.pgph.0004419)
Supplement: Attachment — Submitted filename: PGPH-D-24-00517_R1_reviewed.pdf [file pgph.0004419.s003.pdf]

PLOS Global Public Health

Assessing the Global Implications of the COVID-19 Pandemic on Cervical Cancer Screening Initiatives: A Comprehensive Evaluation

--Manuscript Draft--

Manuscript Number:	PGPH-D-24-00517R1
Article Type:	Research Article
Full Title:	Assessing the Global Implications of the COVID-19 Pandemic on Cervical Cancer Screening Initiatives: A Comprehensive Evaluation
Short Title:	Evaluating the Impact of COVID-19 on Cervical Cancer Screening Programs Across the Globe
Corresponding Author:	Prajakta Adsul, MBBS, MPH, PhD<br>The University of New Mexico Comprehensive Cancer Center: UNM Comprehensive Cancer Center<br>Albuquerque, New Mexico UNITED STATES OF AMERICA
Order of Authors:	Anisha M Loeb, MPH
	Patti Gravitt
	Allison Frank
	Douglas M. Puricelli Perin
	Kalina Duncan
	Linda Eckert
	Maribel Almonte
	Nathalie Broutet
	Joseph Rodman
	Oyeleke A Oyebamiji
	Prajakta Adsul
Keywords:	cervical cancer screening; COVID-19; Implementation science; HPV; health systems research
Abstract:	Introduction<br><br>The COVID-19 pandemic disrupted many public health programs; understanding these disruptions is critical for directing future resources. In a project studying the implementation of human papillomavirus (HPV) testing-based cervical cancer screening, we queried about the impact of the pandemic on screening programs globally.<br><br>Methods<br><br>In consultation with World Health Organization's Regional Advisors, program managers, government officials, and clinicians involved in the implementation of HPV testing-based cervical cancer screening programs were invited to participate in semi-structured, in-depth, interviews. Interview notes and transcripts were used for inductive analysis, focusing on responses to the impact of COVID-19 pandemic on screening programs.<br><br>Results<br><br>Thirty-two interviews were conducted with participants between the age of 29 and 61 years, representing programs from 25 countries. Six key themes were noted. Regarding disruptions, (1) the entire cancer continuum was affected, leading to delays or, in some cases even cessation of vaccination, screening, and treatment programs; and (2) a heightened sense of fear around contracting and transmitting COVID-19

Powered by Editorial Manager® and ProduXion Manager® from Aries Systems Corporation

	shifted government priorities and impacted healthcare delivery. Nonetheless, participants noted constructive ways in which programs leveraged the impact of the pandemic: (1) at the community level, participants were able to leverage an increased understanding and acceptance surrounding the importance of preventive health behaviors; (2) for HPV-testing: molecular laboratories became well-equipped with better technician training, increasing overall HPV-testing capacities; (3) the pandemic promoted virtual healthcare systems; and (4) for planning: shutdowns allowed time to plan for program scale-up. In many ways, the pandemic response provided evidence supporting the country's ability to mobilize resources.<br><br>Conclusions<br><br>While disruptions were noted, the pandemic provided implementers with opportunities to strengthen screening programs, which should be further assessed in future sustainability research on cervical cancer prevention and control.
**Additional Information:**	
**Question**	**Response**
**Financial Disclosure**<br><br>Enter a financial disclosure statement that describes the sources of funding for the work included in this submission and the role the funder(s) played. This includes grants and any commercial funding of the work or authors.<br><br>This statement will be typeset if the manuscript is accepted for publication.<br><br>*Please review the submission guidelines and the instructions link below for detailed requirements and guidance.*	This project has been funded in whole or in part with federal funds from the National Cancer Institute, National Institutes of Health, under Contract No. 75N91019D00024. The content of this publication does not necessarily reflect the views or policies of the Department of Health and Human Services, nor does mention of trade names, commercial products, or organizations imply endorsement by the U.S. Government. In addition, this project has been funded by the UNDP (United Nations Development Program)–UNFPA (United Nations Population Fund)–UNICEF–WHO–World Bank Special Program of Research in Human Reproduction (HRP) (contract to Dr. Adsul; WHO PO # 202833289). The funders had no role in the design of the study and analysis of data.
**Competing Interests**<br><br>On behalf of all authors, disclose any competing interests that could be perceived to bias this work.<br><br>This statement will be typeset if the manuscript is accepted for publication.<br><br>*Please review the instructions link below and PLOS Global Public Health's competing interests policy to determine what information must be disclosed at*	Authors declare no competing interest.

submission.	
**Data Availability**<br><br>Before publication, Authors are required to make fully available and without restriction all data underlying their findings. Please see our PLOS Data Policy page for detailed information on this policy.<br><br>A **Data Availability Statement**, detailing where the data can be accessed, is required at first submission. Insert your Data Availability Statement in the box below.<br><br>Please see the data reporting section of our submission guidelines for instructions on what you need to include in your Data Availability Statement.<br><br>This statement will be typeset if the manuscript is accepted for publication.<br><br>*PLOS allows rare exemptions to address legal and ethical concerns. If you have legal or ethical restrictions, please use the box below to detail these in full sentences for the Journal team to consider.*	All data are provided in the manuscript.

Powered by Editorial Manager® and ProduXion Manager® from Aries Systems Corporation

Manuscript
Click here to access/download;Manuscript;Manuscript_062524.docx

Full Title: Assessing the Global Implications of the COVID-19 Pandemic on Cervical Cancer Screening Initiatives: A Comprehensive Evaluation

Short Title: Evaluating the Impact of COVID-19 on Cervical Cancer Screening Programs Across the Globe

Authors: Anisha M. Loeb[1], Patti Gravitt,[2] Allison Frank,[2] Douglas M. Puricelli Perin,[3] Kalina Duncan,[2] Linda Eckert,[4,5] Maribel Almonte, [6,7,] Nathalie Broutet,[8] Joseph Rodman[9], Oyeleke A Oyebamiji[9], Prajakta Adsul[9,10]

Affiliations:

[1]Department of Epidemiology, School of Public Health, University of Washington, Seattle, WA

[2]Center for Global Health, National Cancer Institute, Rockville, MD

[3] Clinical Monitoring Research Program Directorate, Frederick National Laboratory for Cancer Research, Frederick, MD, USA

[4]Department of Obstetrics & Gynaecology, University of Washington, Seattle, WA

[5]Department of Global Health, School of Public Health, University of Washington, Seattle, WA

[6]Department of Non-Communicable Diseases, World Health Organization, Geneva, Switzerland

[7]Early Detection and Prevention Program, International Agency for Research on Cancer, Lyon, France

[8]Department of Sexual and Reproductive Health and Research, World Health Organization, Geneva, Switzerland

[9]Cancer Control and Population Sciences, University of New Mexico Comprehensive Cancer Center, Albuquerque, NM

[10]Department of Internal Medicine, University of New Mexico, Albuquerque, NM

Funding statement: This project has been funded by the United Nations Development Program - United Nations Population Fund– World Health Organization –World Bank Special Program of Research in Human Reproduction (HRP), to PA (WHO PO # 202833289). In addition, the project received support from the National Cancer Institute, National Institutes of Health, under Contract

No. 75N91019D00024. The content of this publication does not necessarily reflect the views or policies of the Department of Health and Human Services, nor does mention of trade names, commercial products, or organizations imply endorsement by the U.S. Government.

Disclaimer: Where authors are identified as personnel of the International Agency for Research on Cancer or World Health Organization, the authors alone are responsible for the views expressed in this article and they do not necessarily represent the decisions, policy or views of the International Agency for Research on Cancer or World Health Organization.

Abstract:

Introduction: The COVID-19 pandemic disrupted many public health programs; understanding these disruptions is critical for directing future resources. In a project studying the implementation of human papillomavirus (HPV) testing-based cervical cancer screening, we queried about the impact of the pandemic on screening programs globally.

Methods: In consultation with World Health Organization's Regional Advisors, program managers, government officials, and clinicians involved in the implementation of HPV testing-based cervical cancer screening programs were invited to participate in semi-structured, in-depth, interviews. Interview notes and transcripts were used for inductive analysis, focusing on responses to the impact of COVID-19 pandemic on screening programs.

Results: Thirty-two interviews were conducted with participants between the age of 29 and 61 years, representing programs from 25 countries. Six key themes were noted. Regarding disruptions, (1) the entire cancer continuum was affected, leading to delays or, in some cases even cessation of vaccination, screening, and treatment programs; and (2) a heightened sense of fear around contracting and transmitting COVID-19 shifted government priorities and impacted healthcare delivery. Nonetheless, participants noted constructive ways in which programs leveraged the impact of the pandemic: (1) at the community level, participants were able to leverage an increased understanding and acceptance surrounding the importance of preventive health behaviors; (2) for HPV-testing: molecular laboratories became well-equipped with better technician training, increasing overall HPV-testing capacities; (3) the pandemic promoted virtual healthcare systems; and (4) for planning: shutdowns allowed time to plan for program scale-up. In many ways, the pandemic response provided evidence supporting countries' abilities to mobilize resources.

Conclusions: While disruptions were noted, the pandemic provided implementers with opportunities to strengthen screening programs, which should be further assessed in future sustainability research on cervical cancer prevention and control.

Introduction

Cervical cancer (CC) continues to be commonly diagnosed and fatal among women in low- and middle-income countries (LMICs) [1]. However, it is preventable through interventions such as human papillomavirus (HPV) vaccination and regular CC screening followed by treatment of positives. Women are vulnerable to HPV infection in the cervix across their lifespan [2], and it is essential that they are screened regularly accompanied by the recommended follow-up and treatment for those who are found to have screening abnormalities. In 2021, the World Health Organization (WHO) put out a recommendation for the CC elimination agenda, which proposed several screening and treatment algorithms, with a strong focus on using HPV DNA testing as the primary screening test due to its high accuracy [3]. In response, many countries have adopted and, in some cases, implemented HPV DNA screening programs following the WHO recommendations; however, there is very limited information guiding the transition to and implementation of population-based HPV DNA screening and treatment programs.

Moreover, the COVID-19 lockdown in March 2020 caused significant disruptions to health systems worldwide. Due to the surge of COVID-19 cases, many health systems were unable to focus on cancer screening and treatment [4]. This led to programmatic shutdowns, decreasing CC screening rates by 52% globally with reductions in rates of colposcopy and invasive CC diagnoses [5–11]. Public interest in cancer lessened as media coverage highlighted COVID-19 as the sole health threat, and Google search trends showed decreases in searches related to cancer screenings [12].

The COVID-19 pandemic intensified barriers to seeking sexual and reproductive health care. There were severe impacts to financial and human resource availability, including supply chain disruptions, reduced programmatic funding, a shortage of healthcare workers, increased healthcare costs, lower appointment availability, and the inability of patients to afford necessary

medical services [5,13–15]. The lack of resources encouraged physicians to adopt a ‘risk-based approach’ in providing care, prioritizing the most vulnerable patients [14]. However, defining vulnerability became difficult when physicians had to consider the severity of the disease versus the number of life-years that the treatment might save. Additionally, community members lost trust in the healthcare system [14,16]. This distrust coupled with fear of contracting COVID-19 and pre-existing transportation barriers further limited and discouraged patients from seeking healthcare services [13–15,17,18].

At the same time, the pandemic also highlighted potential strategies to enhance healthcare delivery, particularly in relation to CC screening. The reduction of in-person clinic visits has led to a greater acceptance of telemedicine visits among providers and patients, which helps to reduce transportation barriers and scheduling constraints for many patients [14,15,18]. HPV testing allows patients to conduct self-sampling at home, which has expanded service delivery [8,14,18]. Companies that conduct microbiology testing reported increases in production capacity and a rise in the set-up of testing platforms to address COVID-19, which is expected to be useful for expanding HPV testing post-pandemic [19]. To track COVID-19 cases, many countries opted for health registries, that can be linked to national health information systems and be leveraged for other diseases such as CC [8]. Lastly, the pandemic has facilitated strong collaboration and coordination between health and governmental sectors which can aid in policy and financial support for preventive health programs [4,5,17,20].

The study is a part of a larger initiative that aimed to generate practice-based evidence for strategies and approaches that support the implementation of HPV DNA-based screening and treatment programs that are ongoing across the globe guided by the Exploration, Preparation, Implementation, and Sustainment Framework [21]. Since these interviews took place amidst the COVID-19 pandemic, there were significant discussions around the impact of the pandemic on

the CC screening and treatment programs. In this paper, we characterize the impacts of COVID-19 on pre-existing CC screening programs in LMICs using data from the interviews. The purpose of this study is to assess strategies that were used to address the impact of COVID-19 on CC screening programs in LMICs and how they could be leveraged to improve the functioning and preparedness of these programs for continuity and resilience amidst future public health emergencies.

Methods

Recruitment

All study activities were initiated after securing appropriate ethical approvals from the WHO Human Reproduction Program and the University of New Mexico’s Human Research Protections Office [Study ID: 21-398]. After consulting with WHO regional advisors and the study team’s professional network, there were recommendations to connect with 84 practitioners/on-the-ground experts involved in CC screening programs from various countries across the globe. The study team, along with WHO regional advisors, sent email communications to all 84 health practitioners to describe the initiative, request and confirm their interest in participation, and schedule an interview. No participant compensation was offered or provided for this study. Written consent was obtained when the interview was scheduled. If, for some reason, the participant did not have time to complete the written consent, verbal consent was obtained with the interviewer as the witness on the recorded Zoom calls.

Data Collection

Two study team members with implementation science and qualitative research expertise, PA and PG, conducted the semi-structured interviews. The interview questions focused on the effectiveness, sustainability, and scale-up potential of existing CC screening programs[illegible]pecific to the analyses presented in this paper, participants were asked “How has COVID affected the

current implementation efforts? Were there specific changes during the pandemic that you have made in program delivery? Reflecting back on the innovative strategies that you have used for cervical cancer screening, are there changes you've made to the algorithms you used? By necessity you have had to be innovative, how many of those seem like better options to continue?" The Zoom virtual meeting platform was used to conduct the interviews, which lasted one to two hours. Simultaneous translation, which consisted of two live interpreters; one on an English channel with the interviewers and one on a French or Spanish channel with the participant, was provided for non-English speakers. There were a total of three interviews which required these efforts (two in French and one in Spanish). A supplemental notetaker attended each interview to ensure interview guide concordance and assisted the interviewer with relevant probes and clarifications as needed. All interviews were audio-recorded and transcribed verbatim with participant consent. We achieved data saturation at 32 interviews.

Data Analysis

The transcripts were uploaded to Dedoose qualitative software to facilitate review, organization, and analysis. The study team used a grounded theory approach to develop the COVID-19-related codes and analyze the interview transcripts and notes. Grounded theory is an inductive qualitative technique to identify themes that emerge from text [22]. Team members (AL, JR, and PA) trained in qualitative analysis read the transcripts for each interview, and independently coded them using Dedoose. During initial coding, the study team met regularly to compare codes and resolve discrepancies by the third cod[illegible] Team discussions led to the identification of six major themes from the data, and the team generated summary statements and narratives based on these themes. Collectively, these themes represent the impact of COVID-19 on the CC screening and treatment programs and were used to organize the results. Participant responses were de-identified to only include the WHO region and profession. Quotes were selected to represent each theme.

Results

Participants

The study team completed 32 interviews between June and October 2022. Participants were aged between 29-61 years and came from 25 countries including: 10 from the Americas region (AMR), 10 from the Africa region (AFR), four from the Europe region (EUR), one from the Eastern Mediterranean region (EMR), three from the Western Pacific region (WPR), and five from the South-East Asia region (SEAR). Out of the 25 countries, in accordance with the World Bank, two is designated as low-income, 10 as lower-middle income, 11 as higher-middle income, and two as high-income [23]. 87% of included countries have a national cervical cancer screening program and approximately half have existing HPV testing programs, introduced from 2011-2023 [24]. Participant professions included health ministry officials, program managers, clinicians, and epidemiologists.

Theme 1: The COVID-19 pandemic had an impact on healthcare facilities and human resources across the CC continuum

Almost all participants noted that the lockdown measures implemented to combat COVID-19 caused the shutdown of healthcare facilities and public health programs, resulting in a negative impact on preventive services. The rates of HPV vaccination and CC screening rates notably decreased. Participants reported full-scale programmatic shutdowns or delays in pilot programs, setting back progress made towards CC elimination. Interestingly, there were some countries where programs continued uninterrupted by COVID-19. However, participants noted that health systems overall were unprepared for the pandemic. A primary-level health professional from the AMR region noted:

“Because our health system …was improper. I think that, that the worse thing was when we had to stop all the programs, all the prevention programs were stopped and … we took up a bad idea to shut down all the primary care facilities. All, all were shut down at the beginning of COVID-19. So that was crazy because all, all the people were sick at the hospitals and we don't have enough hospitals, but that decision was made and all the programs …that weren't COVID-19 were shut down”

All participants noted that to address the global emergency, financial and human resources were redirected from health programs, negatively impacting the procurement of HPV DNA tests, the availability of trained professionals, and overall testing capacity. This resulted in a backlog in diagnostic testing, which led to a subsequent backlog in treatment referrals. A lead physician in the AFR region noted:

“Outpatient was literally closed, and they turned that area into a COVID-19 area. That meant that the screening availability was also quite reduced. Then there was a time the referrals were stopped.”

Many healthcare workers were mobilized toward COVID-19-related needs, and some were diverted to providing primary care to patients living with the virus. The diversion of these trained professionals impacted the scale and quality of care during the pandemic, but also continued to impact public health programs with the need for refresher trainings. A tertiary-level health professional from the SEAR region, not yet having implemented HPV screening, noted the impacts of COVID-19 on the workforce for VIA:

“So, the main challenges retaining the trained staff in the same program when COVID-19 pandemic stuck. All the people trained for VIA [visual inspection using acetic acid],

> *they are lost. They have been scattered into different health programs, so now getting them back… to the same program by itself is a challenge."*

While HPV testing was still being rolled out in some regions, participants noted that the pandemic resulted in a clear need to collect cervical samples with a limited number of healthcare workers, leading to favoring of HPV DNA tests which allowed self-sampling. Many noted that self-sampling could increase screening rates by reaching more women and many interviewees indicated patient preference towards self-sampling increasing their comfort and eliminating transportation barriers. A program director in the AFR region noted:

> *"Due to COVID-19, we were, I don't want to say forced, but were kind of forced to think through a more innovative way of getting samples without having women come to the facility. This is where we added the self-sampling arm both for the women who are in the facility. They will be given the opportunity to self-sample within the facility. I agree, for me, this is the most cost-effective way of self-sampling because it eliminates first the stigma around a pelvic exam, but it also eliminates the requirement for a speculum which is a challenge in our setting, procuring as many speculums as needed."*

Theme 2: The fear of contracting and transmitting COVID-19 shifted government priorities and impacted healthcare delivery

Several participants pointed out that the success and sustainability of programs depend on governmental or ministry support. During the pandemic, participants stressed that any focus governmental officials had on implementing CC screening programs, as well as human resources, were diverted to COVID-19 testing and care, which significantly impeded the already underfunded public health and cancer prevention programs. A public health specialist in the AFR region noted:

"COVID-19 vaccine campaigns disrupted service delivery at health care facilities and decreased government spending for cervical cancer related services due to competing priorities."

This disruption not only impacted preventive measures during the pandemic as reported by the interviewees but when countries were able to prioritize CC, it was difficult to resume programmatic implementation. Thus, many existing programs were affected for longer than initially thought, and screening and vaccination rates remained consistently low.

Several participants expressed widespread fear about COVID-19 among individuals seeking healthcare as well as frontline workers. A Ministry of Health representative from the AMR region noted:

"So, I think the human resources definitely got hit hard where we had to sacrifice primary care interventions. Fear from COVID-19 was present on the demand side and the supply side. We rushed people away from the health care facilities so that they don't get infected, or they don't infect us."

It was reported that this fear also led to physical distancing protocols, which negatively impacted the delivery of in-person healthcare services as well as community engagement and outreach. Screening rates dropped in populations who especially rely on community campaigns for education and service delivery. A lead physician from the AFR region summarized:

"We had this recommendation that come out for social distancing purposes, you can no longer conduct outreach, especially during the peaks. That actually resulted in reduced screening rates."

Theme 3: Awareness around the COVID-19 pandemic led to an increase in understanding about preventive health services

Almost all participants acknowledged that the COVID-19 pandemic increased public awareness of viruses and how they are transmitted, and that such awareness led to a greater acceptance of preventive health measures such as vaccinations and early detection. A professor from the AMR region highlighted the benefits of this increased awareness and emphasized the need for further messaging to promote screening programs:

> *"I think one of the critical things, I believe that …everybody by the way after COVID-19, understands what a virus is, what a virus can do, and what a molecular test is. That's a big advantage. They understand that somebody can stay with the virus. They even are asking me if they can be vaccinated because they also think about COVID-19. Unfortunately, we have to tell them that we can vaccinate only the girls for the future, but they already had the virus, so at this point what we need to do is to find that the virus is there, and we have to treat them. We're using that message, so communication is also an important thing."*

As noted above, this perceived improvement in awareness among the populations may be a potential advantage for public health communications for CC prevention.

Theme 4: Preparedness for COVID-19 testing had an impact on CC screening testing capacities

According to several participants, the laboratories used the same testing platforms for various diseases such as HPV, HIV, and TB. PCR tests for COVID-19 also leveraged these same testing

platforms and thus countries were able to use pre-existing testing platforms to address the pandemic. This meant that the testing capacity for CC was undercut, which led to delays in testing and increased chances of loss to follow-up. A health authority professional from the AFR region noted:

> *"Oh, COVID-19 testing. Another thing is COVID-19 testing all platforms. Oh my God. That was a nightmare because all platforms were busy with COVID-19 testing. So, all the HPV testing were put on hold for like--in some places, even for two months."*

However, to combat the scale of the pandemic, more individuals received training and became familiar with molecular testing platforms. Additional financial support and improvement of physical infrastructure increased the testing capacities in these laboratories. These advancements in testing capabilities can be leveraged for HPV testing, especially as the incidence of COVID-19 has slowed down. A health authority professional from the AMR region noted, candidly:

> *"But, with the change of COVID-19, biomolecular testing is no longer rocket science. So there's an opportunity to think of other technologies that could be used, and rethink,… what if now we could do this dry swabs... [if] we need a PCR machine and we could do this in a regional hospital...So I think there's an opportunity to use technologies that are not going to be used, hopefully, for SARs, COVID 2 in the future, that are in a lot of these hospitals that had no biomolecular testing in the past. So, that's another opportunity."*

Participants shared many lessons learned from implementing widespread COVID-19 testing that could inform future processes for HPV testing. This includes the use of barcodes to ensure quality,

text messaging to communicate results linked to health information systems, and the centralization of services, as explained by an epidemiologist in the EUR region:

> *"We take one positive advantage that year because that year was many connections with Directory of Public Health of the District with the Institute of Public Health. Because they collected the sample for COVID-19 and they sent all these samples in the lab of the Institute. Because the lab of the Institute of [country name removed] was the main lab for analyzing COVID-19 tests. There were many connections, so this is the positive to cover all the program in a few months, the program of the screening."*

Theme 5: The COVID-19 pandemic revealed fragmentation of healthcare systems and also promoted a move towards virtual healthcare systems

Some participants shared concerns about the impact of the pandemic on the fragmented healthcare systems, while adding that well-functioning systems are crucial for screening programs to remain successful. An epidemiologist from the AMR region highlighted this problem, describing through a scenario of managing patients living with COVID-19 compared to other primary care services:

> *"Okay, if a woman is HPV positive at the center, where will they be sent? To hospital A. Okay, these women were sent to the hospital for that's where they'll get a colposcopy…when hospital A is closed right now. Oh, wait, hospital A is close? Yeah, because of COVID-19. You only take COVID-19 patients? Yes. Where are you going to send the women? To hospital A. But you're saying it's closed. Yes. They're going to be brought to the hospital. They're going to return back. Yes. And then, what? They will have to figure something else out."*

While there were challenges noted in healthcare delivery, many participants noted that community health education programs and professional training programs could be moved to an online delivery mode. Some participants even mentioned they preferred the virtual modality and planned to continue using it after the pandemic due to its reach. Several participants noted that physicians were more accepting of telemedicine appointments, which increased capacity under social-distancing protocols and will continue to do so if patients prefer virtual appointments. A tertiary-level health professional from the WPR region noted:

> *"Yeah. And, especially after COVID-19 because during COVID-19, we carried out many activities only through the live-stream. I mean, even for health counselling are through apps, or through live-streaming on You-Tube, etc. Now, they are more-or-less familiar with that kind of tele-scheduling."*

Theme 6: Additional downtime due to the pandemic was advantageous in the future of programmatic implementation and CC screening potentials

Some participants mentioned that the shutdowns and downtime in implementation efforts allowed them to pivot and at times modify their efforts on targeting specific high-risk groups and planning community campaigns. A lead physician in the AFR region cited:

> *"But what we then did, is we utilized that sort of downtime to plan for sort of rapid scale up post pandemic. Yeah. What we did is to actually try and mobilize resources, find out what is needed, the gloves, do you need to procure more speculums and things like that. Then to also communicate to the respective cadres within the regions that we plan to have a campaign, maybe after three months or so. We had these targets that we*

> *couldn't get. When the COVID-19 restrictions are relaxed, we are going to have a massive campaign, and we need to have massive tracking of patients."*

Moreover, the manner in which countries around the world united and mobilized resources to address COVID-19 demonstrated to the participants the hidden potential to address other global health disparities. Participants reported feeling more confident in improving health outcomes for CC. A tertiary-level health professional from the SEAR region elaborated:

> *"Again, the COVID-19 testing only like gives us confidence. How they centralized. When the government was able to do that COVID-19 testing in such a huge manner in a very short time with limited resources. So, it should be possible for HPV DNA also, so that gives us the confidence."*

Discussion

The emerging healthcare delivery literature has made it abundantly clear that COVID-19 had a substantial impact on health systems across the globe. On-the-ground experts included in this study perceived a shift in government priorities, which resulted in shifts in human and financial resources. Public health initiatives aimed at reducing COVID-19 transmission as well as community fear surrounding an unknown virus effectively decreased the opportunities that individuals had to go into clinics in-person to receive CC preventive services, including vaccination and screening. This effect was reported in a number of studies, who quantified vaccination, screening, and treatment rates, comparing pre-pandemic to mid-pandemic rates [6–10]. Nonetheless, the pandemic presented global healthcare systems with opportunities to find strategies to strengthen preventive services and programs. Community members gained familiarity in modes of viral transmission and ways to protect oneself, which can be used to promote CC preventive services. The laboratories were equipped with PCR machines for COVID-

19 testing and an increasing number of technicians were trained to run these tests. Because these machines are not specific to COVID-19, there exists increased infrastructure for other molecular tests such as HPV testing. Molecular diagnostic companies are also optimistic in the rise of molecular testing potentials, as cited in Poljak et. al [19].

While many health systems revealed poor coordination efforts, there was a need to shift the ways in which some practices were conducted. The rise in virtual activities associated with social distancing protocols may have resulted in increased acceptance and usage of telemedicine and web-delivered trainings for the healthcare professional workforce. HPV DNA tests, in which samples can be collected without the need of a clinician, seemed to gain increased acceptance among healthcare professionals and public health decision-makers as a method to continue screening in times of health emergencies, despite the cost. Numerous studies highlighted the rise of telemedicine and self-sampling as ways that CC preventive efforts continued to function and can increase rates even post-pandemic [8,14,15,18]. The additional time experts had during programmatic holds or shutdowns allowed for increased planning efforts and strategies to target those most at risk. Ultimately, the way the global community mobilized together to tackle COVID-19 proved the ability for health systems to work together to help prevent disease transmission and outbreaks.

Some prior studies mentioned the lack of impact of COVID-19, especially on colposcopy and treatment rates [7–9]. While we did not directly find these results, we did become aware of the differential impact COVID-19 had on regions around the world. For instance, many African countries noted short-term shutdowns of health services. Moreover, studies on high-income countries expressed the inability of patients to afford healthcare services during the pandemic [14]. As our study focused primarily on LMICs, whose populations experience these hardships

under non-pandemic conditions, we did not find increased inability to afford healthcare be a driving consequence of COVID-19.

This study had some limitations, which need to be acknowledged. The study sample was comprised of public health and health care practitioners who responded to email invitations among those selected by WHO regional advisors and study team members based on their expertise, knowledge, and involvement in CC screening programs in their countries. While the initial goal was to obtain representation from each WHO region, some were less responsive than others that may be reflective of overburdened healthcare systems during the pandemic. Thus, study conclusions may not reflect experiences from individuals in countries that were not a part of the study. The data presented were validated through notes taken during interviews and transcripts developed from audio recordings. However, we were unable to conduct participant checking to ensure our understanding of certain points was as the interviewee intended. Lastly, as the pandemic was still a global emergency impacting health systems during the time we conducted the interviews, some responses were theoretical as to how certain systems may change because of COVID-19, and future research could generate the concrete evidence to confirm these hypotheses. Further, the primary focus of our study was on screening services so when participants did comment on vaccination, precancer treatment, and cancer treatment rates, we did not probe to learn more about the impact of COVID-19 on those rates.

The study has many strengths. To the best of our knowledge, this was one of the first studies to gather global in-depth data, using a qualitative method of inquiry, in capturing the practice-based understanding for the implementation of CC screen and treatment programs. We reached a point of data saturation, which means that interviewers and analysts noted repetitions of concepts and ideas presented by the participants. This allowed us to feel confident in drawing inferences about the impact of the COVID-19 pandemic from these data. The semi-structured nature of the in-depth

interviews allowed us to strengthen the interview guide as more interviews were conducted. We interviewed individuals involved in many aspects of CC screening programs, from the research up to the national policy-making level, which allowed us to gain multiple insights and perspectives on the impacts of COVID-19. Our future studies, post-pandemic, will evaluate whether these strategies have been kept in place and whether they have helped to sustain and strengthen CC screening programs across the globe.

Overall, study findings suggest that strategies that helped keep health services functional throughout the pandemic have the potential to be leveraged to improve CC prevention for rapid progress towards CC elimination.

Acknowledgments

We sincerely thank the study participants for their insights and time.

References

1. Singh D, Vignat J, Lorenzoni V, Eslahi M, Ginsburg O, Lauby-Secretan B, et al. Global estimates of incidence and mortality of cervical cancer in 2020: a baseline analysis of the WHO Global Cervical Cancer Elimination Initiative. The Lancet Global Health. 2023 Feb 1;11(2):e197–206.

2. Guo F, Hirth JM, Berenson AB. Comparison of HPV prevalence between HPV-vaccinated and non-vaccinated young adult women (20–26 years). Hum Vaccin Immunother. 2015 Sep 16;11(10):2337–44.

3. WHO guideline for screening and treatment of cervical pre-cancer lesions for cervical cancer prevention: Use of mRNA tests for human papillomavirus (HPV). In: 2nd ed. Geneva: World Health Organization; 2021 [cited 2023 Dec 7]. Available from: https://www.ncbi.nlm.nih.gov/books/NBK576617/

4. Huguet N, Danna M, Baron A, Hall J, Hodes T, O'Malley J, et al. Delivery of Cervical and Colorectal Cancer Screenings During the Pandemic in Community Health Centers. Med Care. 2023 Aug;61(8):554–61.

5. Puricelli Perin DM, Elfström KM, Bulliard JL, Burón A, Campbell C, Flugelman AA, et al. Early assessment of the first wave of the COVID-19 pandemic on cancer screening services: The International Cancer Screening Network COVID-19 survey. Prev Med. 2021 Oct;151:106642.

6. Desta AA, Woldearegay TW, Gebremeskel E, Alemayehu M, Getachew T, Gebregzabiher G, et al. Impacts of COVID-19 on essential health services in Tigray, Northern Ethiopia: A pre-post study. PLOS ONE. 2021 Aug 27;16(8):e0256330.

7. Martins TR, Witkin SS, Mendes-Corrêa MC, de Godoy AS, Cury L, Balancin ML, et al. Impact of the COVID-19 Pandemic on Cervical Cancer Screening in São Paulo State, Brazil. Acta Cytol. 2023 Jan 20;1–7.

8. Cruz-Valdez A, Palacio-Mejía LS, Quezada-Sánchez AD, Hernández-Ávila JE, Galicia-Carmona T, Cetina-Pérez L del C, et al. Cervical cancer prevention program in Mexico disrupted due to COVID-19 pandemic: Challenges and opportunities. Frontiers in Oncology [Internet]. 2023 [cited 2023 Jul 27];13. Available from: https://www.frontiersin.org/articles/10.3389/fonc.2023.1008560

9. Lucas E, Murillo R, Arrossi S, Bárcena M, Chami Y, Nessa A, et al. Quantification of impact of COVID-19 pandemic on cancer screening programmes – a case study from Argentina, Bangladesh, Colombia, Morocco, Sri Lanka, and Thailand. eLife. 12:e86527.

10. Ferrara P, Dallagiacoma G, Alberti F, Gentile L, Bertuccio P, Odone A. Prevention, diagnosis and treatment of cervical cancer: A systematic review of the impact of COVID-19 on patient care. Prev Med. 2022 Nov;164:107264.

11. Lee J, Ismail-Pratt I, Machalek DA, Kumarasamy S, Garland SM. The recovery strategies to support cervical cancer elimination in lower-and middle-income countries (LMICs) following COVID-19 disruptions. Preventive Medicine Reports. 2023 Oct 1;35:102291.

12. Cohen SA, Ebrahimian S, Cohen LE, Tijerina JD. Online public interest in common malignancies and cancer screening during the COVID-19 pandemic in the United States. J Clin Transl Res. 2021 Dec 28;7(6):723–32.

13. Murewanhema G. Reduced cervical cancer screening in Zimbabwe as an indirect impact of the COVID-19 pandemic: implications for prevention. Pan Afr Med J. 2021 Feb 5;38:131.

14. Wentzensen N, Clarke MA, Perkins RB. Impact of COVID-19 on cervical cancer screening: Challenges and opportunities to improving resilience and reduce disparities. Prev Med. 2021 Oct;151:106596.

15. Ng'andu M, Mesic A, Pry J, Mwamba C, Roff F, Chipungu J, et al. Sexual and reproductive health services during outbreaks, epidemics, and pandemics in sub-Saharan Africa: a literature scoping review. Systematic Reviews. 2022 Aug 9;11(1):161.

16. DeBoer RJ, Fadelu TA, Shulman LN, Van Loon K. Applying Lessons Learned From Low-Resource Settings to Prioritize Cancer Care in a Pandemic. JAMA Oncology. 2020 Sep 1;6(9):1429–33.

17. Alfaro K, Soler M, Maza M, Flores M, López L, Rauda JC, et al. Cervical Cancer Prevention in El Salvador: Gains to Date and Challenges for the Future. Cancers (Basel). 2022 Jun 3;14(11):2776.

18. Rodriguez NM, Brennan LP, Claure L, Balian LN, Champion VL, Forman MR. Leveraging COVID-era innovation for cervical cancer screening: Clinician awareness and attitudes toward self-sampling and rapid testing for HPV detection. PLoS One. 2023;18(3):e0282853.

19. Poljak M, Cuschieri K, Waheed DEN, Baay M, Vorsters A. Impact of the COVID-19 pandemic on human papillomavirus-based testing services to support cervical cancer screening. Acta Dermatovenerol Alp Pannonica Adriat. 2021 Mar;30(1):21–6.

20. Puricelli Perin DM, Christensen T, Burón A, Haas JS, Kamineni A, Pashayan N, et al. Interruption of cancer screening services due to COVID-19 pandemic: lessons from previous disasters. Prev Med Rep. 2021 May 17;23:101399.

21. Aarons GA, Hurlburt M, Horwitz SM. Advancing a Conceptual Model of Evidence-Based Practice Implementation in Public Service Sectors. Adm Policy Ment Health. 2011 Jan 1;38(1):4–23.

22. Glaser B, Strauss A. The discovery of grounded theory: strategies for qualitative research. 5th ed. New Brunswick: Aldine Transaction; 2010. 271 p.

23. World Bank Country and Lending Groups [Internet]. 2024 [cited 2024 Jun 1]. Available from: https://datahelpdesk.worldbank.org/knowledgebase/articles/906519-world-bank-country-and-lending-groups

24. Cervical cancer country profiles [Internet]. World Health Organization; 2021 Nov [cited 2024 Jun 1]. Available from: https://www.who.int/teams/noncommunicable-diseases/surveillance/data/cervical-cancer-profiles

Click here to access/download
Supporting Information
Inclusivity_questionnaire.docx

Full Title: Assessing the Global Implications of the COVID-19 Pandemic on Cervical Cancer Screening Initiatives: A Comprehensive Evaluation

Short Title: Evaluating the Impact of COVID-19 on Cervical Cancer Screening Programs Across the Globe

Authors: Anisha M. Loeb[1], Patti Gravitt,[2] Allison Frank,[2] Douglas M. Puricelli Perin,[3] Kalina Duncan,[2] Linda Eckert,[4,5] Maribel Almonte, [6,7,] Nathalie Broutet,[8] Joseph Rodman[9], Oyeleke A Oyebamiji[9], Prajakta Adsul[9,10]

Affiliations:

[1]Department of Epidemiology, School of Public Health, University of Washington, Seattle, WA

[2]Center for Global Health, National Cancer Institute, Rockville, MD

[3] Clinical Monitoring Research Program Directorate, Frederick National Laboratory for Cancer Research, Frederick, MD, USA

[4]Department of Obstetrics & Gynaecology, University of Washington, Seattle, WA

[5]Department of Global Health, School of Public Health, University of Washington, Seattle, WA

[6]Department of Non-Communicable Diseases, World Health Organization, Geneva, Switzerland

[7]Early Detection and Prevention Program, International Agency for Research on Cancer, Lyon, France

[8]Department of Sexual and Reproductive Health and Research, World Health Organization, Geneva, Switzerland

[9]Cancer Control and Population Sciences, University of New Mexico Comprehensive Cancer Center, Albuquerque, NM

[10]Department of Internal Medicine, University of New Mexico, Albuquerque, NM

Funding statement: This project has been funded by the United Nations Development Program - United Nations Population Fund– World Health Organization –World Bank Special Program of Research in Human Reproduction (HRP), to PA (WHO PO # 202833289). In addition, the project received support from the National Cancer Institute, National Institutes of Health, under Contract

No. 75N91019D00024. The content of this publication does not necessarily reflect the views or policies of the Department of Health and Human Services, nor does mention of trade names, commercial products, or organizations imply endorsement by the U.S. Government.~~This project has been funded in whole or in part with federal funds from the National Cancer Institute, National Institutes of Health, under Contract No. 75N91019D00024. The content of this publication does not necessarily reflect the views or policies of the Department of Health and Human Services, nor does mention of trade names, commercial products, or organizations imply endorsement by the U.S. Government. In addition, this project has been funded by the UNDP (United Nations Development Program)–UNFPA (United Nations Population Fund)–UNICEF–WHO–World Bank Special Program of Research in Human Reproduction (HRP) (contract to Dr. Adsul; WHO PO # 202833289).~~

Disclaimer: Where authors are identified as personnel of the International Agency for Research on Cancer or World Health Organization, the authors alone are responsible for the views expressed in this article and they do not necessarily represent the decisions, policy or views of the International Agency for Research on Cancer or World Health Organization.

Abstract:

Introduction: The COVID-19 pandemic disrupted many public health programs; understanding these disruptions is critical for directing future resources. In a project studying the implementation of human papillomavirus (HPV) testing-based cervical cancer screening, we queried about the impact of the pandemic on screening programs globally.

Methods: In consultation with World Health Organization's Regional Advisors, program managers, government officials, and clinicians involved in the implementation of HPV testing-based cervical cancer screening programs were invited to participate in semi-structured, in-depth, interviews. Interview notes and transcripts were used for inductive analysis, focusing on responses to the impact of COVID-19 pandemic on screening programs.

Results: Thirty-two interviews were conducted with participants between the age of 29 and 61 years, representing programs from 25 countries. Six key themes were noted. Regarding disruptions, (1) the entire cancer continuum was affected, leading to delays or, in some cases even cessation of vaccination, screening, and treatment programs; and (2) a heightened sense of fear around contracting and transmitting COVID-19 shifted government priorities and impacted healthcare delivery. Nonetheless, participants noted constructive ways in which programs leveraged the impact of the pandemic: (1) at the community level, participants were able to leverage an increased understanding and acceptance surrounding the importance of preventive health behaviors; (2) for HPV-testing: molecular laboratories became well-equipped with better technician training, increasing overall HPV-testing capacities; (3) the pandemic promoted virtual healthcare systems; and (4) for planning: shutdowns allowed time to plan for program scale-up. In many ways, the pandemic response provided evidence supporting ~~the~~ countries'~~y's~~ abilities~~y~~ to mobilize resources.

Conclusions: While disruptions were noted, the pandemic provided implementers with opportunities to strengthen screening programs, which should be further assessed in future sustainability research on cervical cancer prevention and control.

Introduction

Cervical cancer (CC) continues to be commonly diagnosed and fatal among women in low- and middle-income countries (LMICs) [1]. However, it is preventable through interventions such as human papillomavirus (HPV) vaccination and regular CC screening followed by treatment of positives. Women are vulnerable to HPV infection in the cervix across their lifespan [2], and it is essential that they are screened regularly accompanied by the recommended follow-up and treatment for those who are found to have screening abnormalities. In 2021, the World Health Organization (WHO) put out a recommendation for the CC elimination agenda, which proposed several screening and treatment algorithms, with a strong focus on using HPV DNA testing as the primary screening test due to its high accuracy [3]. In response, many countries have adopted and, in some cases, implemented HPV DNA screening programs following the WHO recommendations; however, there is very limited information guiding the transition to and implementation of population-based HPV DNA screening and treatment programs.

Moreover, the COVID-19 lockdown in March 2020 caused significant disruptions to health systems worldwide. Due to the surge of COVID-19 cases, many health systems were unable to focus on cancer screening and treatment [4]. This led to programmatic shutdowns, decreasing CC screening rates by 52% globally with reductions in rates of colposcopy and invasive CC diagnoses [5–11]. Public interest in cancer lessened as media coverage highlighted COVID-19 as the sole health threat, and Google search trends showed decreases in searches related to cancer screenings [12].

The COVID-19 pandemic intensified barriers to seeking sexual and reproductive health care. There were severe impacts to financial and human resource availability, including supply chain disruptions, reduced programmatic funding, a shortage of healthcare workers, increased healthcare costs, lower appointment availability, and the inability of patients to afford necessary

medical services [5,13–15]. The lack of resources encouraged physicians to adopt a ‘risk-based approach’ in providing care, prioritizing the most vulnerable patients [14]. However, defining vulnerability became difficult when physicians had to consider the severity of the disease versus the number of life-years that the treatment might save. Additionally, community members lost trust in the healthcare system [14,16]. This distrust coupled with fear of contracting COVID-19 and pre-existing transportation barriers further limited and discouraged patients from seeking healthcare services [13–15,17,18].

At the same time, the pandemic also highlighted potential strategies to enhance healthcare delivery, particularly in relation to CC screening. The reduction of in-person clinic visits has led to a greater acceptance of telemedicine visits among providers and patients, which helps to reduce transportation barriers and scheduling constraints for many patients [14,15,18]. HPV testing allows patients to conduct self-sampling at home, which has expanded service delivery [8,14,18]. Companies that conduct microbiology testing reported increases in production capacity and a rise in the set-up of testing platforms to address COVID-19, which is expected to be useful for expanding HPV testing post-pandemic [19]. To track COVID-19 cases, many countries opted for health registries, that can be linked to national health information systems and be leveraged for other diseases such as CC [8]. Lastly, the pandemic has facilitated strong collaboration and coordination between health and governmental sectors, which can aid in policy and financial support for preventive health programs [4,5,17,20].

The study is a part of a larger initiative that aimed to generate practice-based evidence for strategies and approaches that support the implementation of HPV DNA-based screening and treatment programs that are ongoing across the globe, guided by the Exploration, Preparation, Implementation, and Sustainment Framework [21]. Since these interviews took place amidst the COVID-19 pandemic, there were significant discussions around the impact of the pandemic on

the CC screening and treatment programs. In this paper, we characterize the impacts of COVID-19 on pre-existing CC screening programs in LMICs using data from the interviews. The purpose of this study is to assess strategies that were used to address the impact of COVID-19 on CC screening programs in LMICs and how they could be leveraged to improve the functioning and preparedness of these programs for continuity and resilience amidst future public health emergencies.

Methods

Recruitment

All study activities were initiated after securing appropriate ethical approvals from the WHO Human Reproduction Program and the University of New Mexico's Human Research Protections Office [Study ID: 21-398]. After consulting with WHO regional advisors and the study team's professional network, there were recommendations to connect with 84 practitioners/on-the-ground experts involved in CC screening programs from various countries across the globe. The study team, along with WHO regional advisors, sent email communications to all 84 health practitioners to describe the initiative, request and confirm their interest in participation, and schedule an interview. No participant compensation was offered or provided for this study. Written consent was obtained when the interview was scheduled. If, for some reason, the participant did not have time to complete the written consent, verbal consent was obtained with the interviewer as the witness on the recorded Zoom calls.

Data Collection

Two study team members with implementation science and qualitative research expertise, PA and PG, conducted the semi-structured interviews. The interview questions focused on the effectiveness, sustainability, and scale-up potential of existing CC screening programs. Specific to the analyses presented in this paper, participants were asked "How has COVID affected the

current implementation efforts? Were there specific changes during the pandemic that you have made in program delivery? Reflecting back on the innovative strategies that you have used for cervical cancer screening, are there changes you’ve made to the algorithms you used? By necessity you have had to be innovative, how many of those seem like better options to continue?”

The Zoom virtual meeting platform was used to conduct the interviews, which lasted one to two hours. Simultaneous translation, which consisted of two live interpreters; one on an English channel with the interviewers and one on a French or Spanish channel with the participant, was provided for non-English speakers. There were a total of three interviews which required these efforts (two in French and one in Spanish). A supplemental notetaker attended each interview to ensure interview guide concordance and assisted the interviewer with relevant probes and clarifications as needed. All interviews were audio-recorded and transcribed verbatim with participant consent. We achieved data saturation at 32 interviews.

Data Analysis

The transcripts were uploaded to Dedoose qualitative software to facilitate review, organization, and analysis. The study team used a grounded theory approach to develop the COVID-19-related codes and analyze the interview transcripts and notes. Grounded theory is an inductive qualitative technique to identify themes that emerge from text [22]. Team members (AL, JR, and PA) trained in qualitative analysis read the transcripts for each interview, and independently coded them using Dedoose. During initial coding, the study team met regularly to compare codes and resolve discrepancies~~,~~ by the third coder. Team discussions led to the identification of six major themes from the data, and the team generated summary statements and narratives based on these themes. Collectively, these themes represent the impact of COVID-19 on the CC screening and treatment programs and were used to organize the results. Participant responses were de-identified to only include the WHO region and profession. Quotes were selected to represent each theme.

Results

Participants

The study team completed 32 interviews between June and October 2022. Participants were aged between 29-61 years and came from 25 countries including: 10 from the Americas region (AMR), 10 from the Africa region (AFR), four from the Europe region (EUR), one from the Eastern Mediterranean region (EMR), three from the Western Pacific region (WPR), and five from the South-East Asia region (SEAR). Out of the 25 countries, in accordance with the World Bank, two is designated as low-income, 10 as lower-middle income, 11 as higher-middle income, and two as high-income [23]. 87% of included countries have a national cervical cancer screening program and approximately half have existing HPV testing programs, introduced from 2011-2023 [24]. Participant professions included health ministry officials~~, clinicians~~, program managers, clinicians, and epidemiologists.

Theme 1: The COVID-19 pandemic had an impact on healthcare facilities and human resources across the CC continuum

Almost all participants noted that the lockdown measures implemented to combat COVID-19 caused the shutdown of healthcare facilities and public health programs, resulting in a negative impact on preventive services. The rates of HPV vaccination and CC screening rates notably decreased. Participants reported full-scale programmatic shutdowns or delays in pilot programs, setting back progress made towards CC elimination. Interestingly, there were some countries where programs continued uninterrupted by COVID-19. However, participants noted that health systems overall were unprepared for the pandemic. A primary-level health professional from the AMR region noted:

“Because our health system …was improper. I think that, that the worse thing was when we had to stop all the programs, all the prevention programs were stopped and … we took up a bad idea to shut down all the primary care facilities. All, all were shut down at the beginning of COVID-19. So that was crazy because all, all the people were sick at the hospitals and we don't have enough hospitals, but that decision was made and all the programs …that weren't COVID-19 were shut down”

All participants noted that to address the global emergency, financial and human resources were redirected from health programs, negatively impacting the procurement of HPV DNA tests, the availability of trained professionals, and overall testing capacity. This resulted in a backlog in diagnostic testing, which led to a subsequent backlog in treatment referrals. A lead physician in the AFR region noted:

“Outpatient was literally closed, and they turned that area into a COVID-19 area. That meant that the screening availability was also quite reduced. Then there was a time the referrals were stopped.”

Many healthcare workers were mobilized toward COVID-19-related needs, and some were diverted to providing primary care to patients living with the virus. The diversion of these trained professionals impacted the scale and quality of care during the pandemic, but also continued to impact public health programs with the need for refresher trainings. A tertiary-level health professional from the SEAR region, not yet having implemented HPV screening, noted the impacts of COVID-19 on the workforce for VIA:

“So, the main challenges retaining the trained staff in the same program when COVID-19 pandemic stuck. All the people trained for VIA [visual inspection using acetic acid],

> *they are lost. They have been scattered into different health programs, so now getting them back… to the same program by itself is a challenge.”*

While HPV testing was still being rolled out in some regions, p~~P~~articipants noted that the pandemic resulted in a clear need to collect cervical samples with a limited number of healthcare workers, leading to favoring of HPV DNA tests which allowed self-sampling. Many noted that self-sampling could increase screening rates by reaching more women and many interviewees indicated patient preference towards self-sampling increasing their comfort and eliminating transportation barriers. A program director in the AFR region noted:

> *“Due to COVID-19, we were, I don’t want to say forced, but were kind of forced to think through a more innovative way of getting samples without having women come to the facility. This is where we added the self-sampling arm both for the women who are in the facility. They will be given the opportunity to self-sample within the facility. I agree, for me, this is the most cost-effective way of self-sampling because it eliminates first the stigma around a pelvic exam, but it also eliminates the requirement for a speculum which is a challenge in our setting, procuring as many speculums as needed.”*

Theme 2: The fear of contracting and transmitting COVID-19 shifted government priorities and impacted healthcare delivery

Several participants pointed out that the success and sustainability of programs depend on governmental or ministry support. During the pandemic, participants stressed that any focus governmental officials had on implementing CC screening programs, as well as human resources, were diverted to COVID-19 testing and care, which significantly impeded the already ~~poorly-~~underfunded public health and cancer prevention programs. A public health specialist in the AFR region noted:

"COVID-19 vaccine campaigns disrupted service delivery at health care facilities and decreased government spending for cervical cancer related services due to competing priorities."

This disruption not only impacted preventive measures during the pandemic as reported by the interviewees, but when countries were able to prioritize CC, it was difficult to resume programmatic implementation. Thus, many existing programs were affected for longer than initially thought, and screening and vaccination rates remained consistently low.

Several participants expressed widespread fear about COVID-19 among individuals seeking healthcare as well as frontline workers. A Ministry of Health representative from the AMR region noted:

"So, I think the human resources definitely got hit hard where we had to sacrifice primary care interventions. Fear from COVID-19 was present on the demand side and the supply side. We rushed people away from the health care facilities so that they don't get infected, or they don't infect us."

It was reported that this fear also led to physical distancing protocols, which negatively impacted the delivery of in-person healthcare services as well as community engagement and outreach. Screening rates dropped in populations who especially rely on community campaigns for education and service delivery. A lead physician from the AFR region summarized:

"We had this recommendation that come out for social distancing purposes, you can no longer conduct outreach, especially during the peaks. That actually resulted in reduced screening rates."

Theme 3: Awareness around the COVID-19 pandemic led to an increase in understanding about preventive health services

Almost all participants acknowledged that the COVID-19 pandemic increased public awareness of viruses and how they are transmitted, and that such awareness led to a greater acceptance of preventive health measures such as vaccinations and early detection. A professor from the AMR region highlighted the benefits of this increased awareness and emphasized the need for further messaging to promote screening programs:

"I think one of the critical things, I believe that …everybody by the way after COVID-19, understands what a virus is, what a virus can do, and what a molecular test is. That's a big advantage. They understand that somebody can stay with the virus. They even are asking me if they can be vaccinated because they also think about COVID-19. Unfortunately, we have to tell them that we can vaccinate only the girls for the future, but they already had the virus, so at this point what we need to do is to find that the virus is there, and we have to treat them. We're using that message, so communication is also an important thing."

As noted above, this perceived improvement in awareness among the populations ~~was an important~~may be a potential advantage for public health communications for CC prevention.

Theme 4: Preparedness for COVID-19 testing had an impact on CC screening testing capacities

According to several participants, the laboratories used the same testing platforms for various diseases such as HPV, HIV, and TB. PCR tests for COVID-19 also leveraged these same testing platforms and thus countries were able to use pre-existing testing platforms to address the pandemic. This meant that the testing capacity for CC was undercut, which led to delays in testing and increased chances of loss to follow-up. A health authority professional from the AFR region noted:

> *"Oh, COVID-19 testing. Another thing is COVID-19 testing all platforms. Oh my God. That was a nightmare because all platforms were busy with COVID-19 testing. So, all the HPV testing were put on hold for like--in some places, even for two months."*

However, to combat the scale of the pandemic, more individuals received training and became familiar with molecular testing platforms. Additional financial support and improvement of physical infrastructure increased the testing capacities in these laboratories. These advancements in testing capabilities can be leveraged for HPV testing, especially as ~~when~~ the incidence of COVID-19 has slowed~~s~~ down. A health authority professional from the AMR region noted, candidly:

> *"But, with the change of COVID-19, biomolecular testing is no longer rocket science. So there's an opportunity to think of other technologies that could be used, and rethink,… what if now we could do this dry swabs... [if] we need a PCR machine and we could do this in a regional hospital...So I think there's an opportunity to use technologies that are not going to be used, hopefully, for SARs, COVID 2 in the future, that are in a lot of these hospitals that had no biomolecular testing in the past. So, that's another opportunity."*

Participants shared many lessons learned from implementing widespread COVID-19 testing that could inform future processes for HPV testing. This includes the use of barcodes to ensure quality, text messaging to communicate results linked to health information systems, and the centralization of services, as explained by an epidemiologist in the EUR region:

> *"We take one positive advantage that year because that year was many connections with Directory of Public Health of the District with the Institute of Public Health. Because they collected the sample for COVID-19 and they sent all these samples in the lab of the Institute. Because the lab of the Institute of [country name removed] was the main lab for analyzing COVID-19 tests. There were many connections, so this is the positive to cover all the program in a few months, the program of the screening."*

Theme 5: The COVID-19 pandemic revealed fragmentation of healthcare systems and also promoted a move towards virtual healthcare systems

Some participants shared concerns about the impact of the pandemic on the fragmented healthcare systems, ~~which~~ while adding that well-functioning systems are crucial for screening programs to remain successful. An epidemiologist from the AMR region highlighted this problem, describing through a scenario of managing patients living with COVID-19 compared to other primary care services:

> *"Okay, if a woman is HPV positive at the center, where will they be sent? To hospital A. Okay, these women were sent to the hospital for that's where they'll get a colposcopy…when hospital A is closed right now. Oh, wait, hospital A is close? Yeah, because of COVID-19. You only take COVID-19 patients? Yes. Where are you going to send the women? To hospital A. But you're saying it's closed. Yes. They're going to be*

> *brought to the hospital. They’re going to return back. Yes. And then, what? They will have to figure something else out.”*

While there were challenges noted in healthcare delivery, many participants noted that community health education programs and professional training programs could be moved to an online delivery mode. Some participants even mentioned they preferred the virtual modality and planned to continue using it after the pandemic, due to its reach. Several participants noted that physicians were more accepting of telemedicine appointments, which increased capacity under social-distancing protocols and will continue to do so if patients prefer virtual appointments. A tertiary-level health professional from the WPR region noted:

> *“Yeah. And, especially after COVID-19 because during COVID-19, we carried out many activities only through the live-stream. I mean, even for health counselling are through apps, or through live-streaming on You-Tube, etc. Now, they are more-or-less familiar with that kind of tele-scheduling.”*

Theme 6: Additional downtime due to the pandemic was advantageous in the future of programmatic implementation and CC screening potentials

Some participants mentioned that the shutdowns and downtime in implementation efforts allowed them to pivot and at times modify their efforts on targeting specific high-risk groups and planning community campaigns. A lead physician in the AFR region cited:

> *“But what we then did, is we utilized that sort of downtime to plan for sort of rapid scale up post pandemic. Yeah. What we did is to actually try and mobilize resources, find out what is needed, the gloves, do you need to procure more speculums and things like that.*

> *Then to also communicate to the respective cadres within the regions that we plan to have a campaign, maybe after three months or so. We had these targets that we couldn't get. When the COVID-19 restrictions are relaxed, we are going to have a massive campaign, and we need to have massive tracking of patients."*

Moreover, the manner in which countries around the world united and mobilized resources to address COVID-19 demonstrated to the participants, the hidden potential to address other global health disparities. Participants reported feeling more confident in improving health outcomes for CC. A tertiary-level health professional from the SEAR region elaborated:

> *"Again, the COVID-19 testing only like gives us confidence. How they centralized. When the government was able to do that COVID-19 testing in such a huge manner in a very short time with limited resources. So, it should be possible for HPV DNA also, so that gives us the confidence."*

Discussion

The emerging healthcare delivery literature has made it abundantly clear that COVID-19 had a substantial impact on health systems across the globe. On-the-ground experts included in this study perceived a shift in government priorities, which resulted in shifts in human and financial resources. Public health initiatives aimed at reducing COVID-19 transmission as well as community fear surrounding an unknown virus effectively decreased the opportunities that individuals had to go into clinics in-person to receive CC preventive services, including vaccination and screening. This effect was reported in a number of studies, who quantified vaccination, screening, and treatment rates, comparing pre-pandemic to mid-pandemic rates [6–10]. Nonetheless, the pandemic presented global healthcare systems with opportunities to find strategies to strengthen preventive services and programs. Community members gained

familiarity in modes of viral transmission and ways to protect oneself, which can be used to promote CC preventive services. The laboratories were equipped with PCR machines for COVID-19 testing and an increasing number of technicians were trained to run these tests. Because these machines are not specific to COVID-19, there exists increased infrastructure for other molecular tests such as HPV testing. Molecular diagnostic companies are also optimistic in the rise of molecular testing potentials, as cited in Poljak et. al [19].

While many health systems revealed poor coordination efforts, there was a need to shift the ways in which some practices were conducted. The rise in virtual activities associated with social distancing protocols may have resulted in increased acceptance and usage of telemedicine and web-delivered trainings for the healthcare professional workforce. HPV DNA tests, in which samples can be collected without the need of a clinician, seemed to gain~~ed~~ increased acceptance among healthcare professionals and public health decision-makers as a method to continue screening in times of health emergencies, despite the cost. Numerous studies highlighted the rise of telemedicine and self-sampling as ways that CC preventive efforts continued to function and can increase rates even post-pandemic [8,14,15,18]. The additional time experts had during programmatic holds or shutdowns allowed for increased planning efforts and strategies to target those most at risk. Ultimately, the way the global community mobilized together to tackle COVID-19~~,~~ proved the ability for health systems to work together to help prevent disease transmission and outbreaks.

Some prior studies mentioned the lack of impact of COVID-19, especially on colposcopy and treatment rates [7–9]. While we did not directly find these results, we did become aware of the differential impact COVID-19 had on regions around the world. For instance, many African countries noted short-term shutdowns of health services. Moreover, studies on high-income countries expressed the inability of patients to afford healthcare services during the pandemic

[14]. As our study focused primarily on LMICs, whose populations experience these hardships under non-pandemic conditions, we did not find increased inability to afford healthcare be a driving consequence of COVID-19.

This study had some limitations, which need to be acknowledged. The study sample was comprised of public health and health care practitioners who responded to email invitations among those selected by WHO regional advisors and study team members based on their expertise, knowledge, and involvement in CC screening programs in their countries. While the initial goal was to obtain representation from each WHO region, some were less responsive than others that maybe reflective of overburdened healthcare systems during the pandemic. Thus, study conclusions may not reflect experiences from individuals in countries that were not a part of the study. The data presented were validated through notes taken during interviews and transcripts developed from audio recordings. However, we were unable to conduct participant checking to ensure our understanding of certain points was as the interviewee intended. Lastly, as the pandemic was still a global emergency impacting health systems during the time we conducted the interviews, some responses were theoretical as to how certain systems may change because of COVID-19, and future research could generate the concrete evidence to confirm these hypotheses. Further, the primary focus of our study was on screening services so when participants did comment on vaccination, precancer treatment, and cancer treatment rates, we did not probe to learn more about the impact of COVID-19 on those rates.

The study has many strengths. To the best of our knowledge, this was one of the first studies to gather global in-depth data, using a qualitative method of inquiry, in capturing the practice-based understanding for the implementation of CC screen and treatment programs. We reached a point of data saturation, which means that interviewers and analysts noted repetitions of concepts and ideas presented by the participants. This allowed us to feel confident in drawing inferences about

the impact of the COVID-19 pandemic from these data. The semi-structured nature of the in-depth interviews allowed us to strengthen the interview guide as more interviews were conducted. We interviewed individuals involved in many aspects of CC screening programs, from the research up to the national policy-making level, which allowed us to gain multiple insights and perspectives on the impacts of COVID-19. Our future studies, post-pandemic, will evaluate whether these strategies have been kept in place and whether they have helped to sustain and strengthen CC screening programs across the globe.

Overall, study findings suggest that strategies that helped keep health services functional throughout the pandemic have the potential to be leveraged to improve CC prevention for rapid progress towards CC elimination.

Acknowledgments

We sincerely thank the study participants for their insights and time.

References

1. Singh D, Vignat J, Lorenzoni V, Eslahi M, Ginsburg O, Lauby-Secretan B, et al. Global estimates of incidence and mortality of cervical cancer in 2020: a baseline analysis of the WHO Global Cervical Cancer Elimination Initiative. The Lancet Global Health. 2023 Feb 1;11(2):e197–206.

2. Guo F, Hirth JM, Berenson AB. Comparison of HPV prevalence between HPV-vaccinated and non-vaccinated young adult women (20–26 years). Hum Vaccin Immunother. 2015 Sep 16;11(10):2337–44.

3. WHO guideline for screening and treatment of cervical pre-cancer lesions for cervical cancer prevention: Use of mRNA tests for human papillomavirus (HPV). In: 2nd ed. Geneva: World Health Organization; 2021 [cited 2023 Dec 7]. Available from: https://www.ncbi.nlm.nih.gov/books/NBK576617/

4. Huguet N, Danna M, Baron A, Hall J, Hodes T, O'Malley J, et al. Delivery of Cervical and Colorectal Cancer Screenings During the Pandemic in Community Health Centers. Med Care. 2023 Aug;61(8):554–61.

5. Puricelli Perin DM, Elfström KM, Bulliard JL, Burón A, Campbell C, Flugelman AA, et al. Early assessment of the first wave of the COVID-19 pandemic on cancer screening services: The International Cancer Screening Network COVID-19 survey. Prev Med. 2021 Oct;151:106642.

6. Desta AA, Woldearegay TW, Gebremeskel E, Alemayehu M, Getachew T, Gebregzabiher G, et al. Impacts of COVID-19 on essential health services in Tigray, Northern Ethiopia: A pre-post study. PLOS ONE. 2021 Aug 27;16(8):e0256330.

7. Martins TR, Witkin SS, Mendes-Corrêa MC, de Godoy AS, Cury L, Balancin ML, et al. Impact of the COVID-19 Pandemic on Cervical Cancer Screening in São Paulo State, Brazil. Acta Cytol. 2023 Jan 20;1–7.

8. Cruz-Valdez A, Palacio-Mejía LS, Quezada-Sánchez AD, Hernández-Ávila JE, Galicia-Carmona T, Cetina-Pérez L del C, et al. Cervical cancer prevention program in Mexico disrupted due to COVID-19 pandemic: Challenges and opportunities. Frontiers in Oncology [Internet]. 2023 [cited 2023 Jul 27];13. Available from: https://www.frontiersin.org/articles/10.3389/fonc.2023.1008560

9. Lucas E, Murillo R, Arrossi S, Bárcena M, Chami Y, Nessa A, et al. Quantification of impact of COVID-19 pandemic on cancer screening programmes – a case study from Argentina, Bangladesh, Colombia, Morocco, Sri Lanka, and Thailand. eLife. 12:e86527.

10. Ferrara P, Dallagiacoma G, Alberti F, Gentile L, Bertuccio P, Odone A. Prevention, diagnosis and treatment of cervical cancer: A systematic review of the impact of COVID-19 on patient care. Prev Med. 2022 Nov;164:107264.

11. Lee J, Ismail-Pratt I, Machalek DA, Kumarasamy S, Garland SM. The recovery strategies to support cervical cancer elimination in lower-and middle-income countries (LMICs) following COVID-19 disruptions. Preventive Medicine Reports. 2023 Oct 1;35:102291.

12. Cohen SA, Ebrahimian S, Cohen LE, Tijerina JD. Online public interest in common malignancies and cancer screening during the COVID-19 pandemic in the United States. J Clin Transl Res. 2021 Dec 28;7(6):723–32.

13. Murewanhema G. Reduced cervical cancer screening in Zimbabwe as an indirect impact of the COVID-19 pandemic: implications for prevention. Pan Afr Med J. 2021 Feb 5;38:131.

14. Wentzensen N, Clarke MA, Perkins RB. Impact of COVID-19 on cervical cancer screening: Challenges and opportunities to improving resilience and reduce disparities. Prev Med. 2021 Oct;151:106596.

15. Ng'andu M, Mesic A, Pry J, Mwamba C, Roff F, Chipungu J, et al. Sexual and reproductive health services during outbreaks, epidemics, and pandemics in sub-Saharan Africa: a literature scoping review. Systematic Reviews. 2022 Aug 9;11(1):161.

16. DeBoer RJ, Fadelu TA, Shulman LN, Van Loon K. Applying Lessons Learned From Low-Resource Settings to Prioritize Cancer Care in a Pandemic. JAMA Oncology. 2020 Sep 1;6(9):1429–33.

17. Alfaro K, Soler M, Maza M, Flores M, López L, Rauda JC, et al. Cervical Cancer Prevention in El Salvador: Gains to Date and Challenges for the Future. Cancers (Basel). 2022 Jun 3;14(11):2776.

18. Rodriguez NM, Brennan LP, Claure L, Balian LN, Champion VL, Forman MR. Leveraging COVID-era innovation for cervical cancer screening: Clinician awareness and attitudes toward self-sampling and rapid testing for HPV detection. PLoS One. 2023;18(3):e0282853.

19. Poljak M, Cuschieri K, Waheed DEN, Baay M, Vorsters A. Impact of the COVID-19 pandemic on human papillomavirus-based testing services to support cervical cancer screening. Acta Dermatovenerol Alp Pannonica Adriat. 2021 Mar;30(1):21–6.

20. Puricelli Perin DM, Christensen T, Burón A, Haas JS, Kamineni A, Pashayan N, et al. Interruption of cancer screening services due to COVID-19 pandemic: lessons from previous disasters. Prev Med Rep. 2021 May 17;23:101399.

21. Aarons GA, Hurlburt M, Horwitz SM. Advancing a Conceptual Model of Evidence-Based Practice Implementation in Public Service Sectors. Adm Policy Ment Health. 2011 Jan 1;38(1):4–23.

22. Glaser B, Strauss A. The discovery of grounded theory: strategies for qualitative research. 5th ed. New Brunswick: Aldine Transaction; 2010. 271 p.

23. World Bank Country and Lending Groups [Internet]. 2024 [cited 2024 Jun 1]. Available from: https://datahelpdesk.worldbank.org/knowledgebase/articles/906519-world-bank-country-and-lending-groups

24. Cervical cancer country profiles [Internet]. World Health Organization; 2021 Nov [cited 2024 Jun 1]. Available from: https://www.who.int/teams/noncommunicable-diseases/surveillance/data/cervical-cancer-profiles

Response for technical comments dated Jul 1, 2024

1. Thank you for providing the requested questionnaire for global inclusivity. However, we note that the questionnaire has been submitted as an ""other"" file type. Kindly resubmit the global inclusivity questionnaire as Supporting file

> **Response**: We have now uploaded the inclusivity questionnaire as a Supporting file.

2. PLOS journals require authors to make all data necessary to replicate their study's findings publicly available without restriction at the time of publication. When specific legal or ethical restrictions prohibit public sharing of a data set, authors must indicate how others may obtain access to the data.

In line with our goal of ensuring long-term data availability to all interested researchers, PLOS' Data Policy states that authors cannot be the sole named individuals responsible for ensuring data access (http://journals.plos.org/plosone/s/data-availability#loc-acceptable-data-sharing-methods). As you have noted, however, your institution does not currently have a point of contact for fielding requests for sensitive data. In light of this, please consider whether any of the following would be possible:

> a) Can you make a de-identified, anonymized, or aggregated data set publicly available in a repository or, if that is not possible, in your manuscript's Supporting Information files? For a list of recommended repositories, some of which are able to hold sensitive data, see here: https://journals.plos.org/plosone/s/recommended-repositories
>
>> **Response:** Please note that in previous responses we have clarified that all data from the study are being included in the manuscript itself. There is no additional data related to this manuscript and we are not requesting any exemptions in this regard. We have updated the online submission form accordingly.
>
> b) Are there any institutional representatives who did not collaborate in the study and are not listed as authors on the manuscript who would be able to hold the data and respond to external requests for data access? If so, please provide their contact information (i.e., email address). Please also provide details on how you will ensure persistent or long-term data storage and availability. For instance, data might be stored in two independent locations.
>
>> **Response:** Since we are not requesting any exemptions in this regard and are not storing any additional data in the repository, we do not believe an institutional representative is needed to provide access to the data. All data for this study are included within the manuscript. We have updated the online submission form accordingly.

Response for technical comments dated 9 June, 2024

Dear Editor,

Thank you for detailing further requirements for submission of our manuscript entitled "Assessing the Global Implications of the COVID-19 Pandemic on Cervical Cancer Screening Initiatives: A Comprehensive Evaluation". Please see the below response to your inquiries.

1. In the online submission form, you indicated that [Insert text from online submission form here].

   All PLOS journals now require all data underlying the findings described in their manuscript to be freely available to other researchers, either
   1. In a public repository,
   2. Within the manuscript itself, or
   3. Uploaded as supplementary information.

   This policy applies to all data except where public deposition would breach compliance with the protocol approved by your research ethics board. If your data cannot be made publicly available for ethical or legal reasons (e.g., public availability would compromise patient privacy), please explain your reasons by return email and your exemption request will be escalated to the editor for approval. Your exemption request will be handled independently and will not hold up the peer review process, but will need to be resolved should your manuscript be accepted for publication. One of the Editorial team will then be in touch if there are any issues."

   > **Response:** Thank you for bringing this to our attention. All data pertinent to the question being answered in this paper are included within the manuscript itself. Additional contextual information can be obtained by contacting the corresponding author.

2. Please remove the old version of the manuscript file and retain only the revised version.

   > **Response:** In the re-submission, we have included a revised version of the manuscript including track changes as well as a version with the changes accepted.

3. Please send a completed 'Competing Interests' statement, including any COIs declared by your co-authors. If you have no competing interests to declare, please state "The authors have declared that no competing interests exist".

   > **Response:** The authors have declared that no competing interests exist.

4. Please amend your detailed Financial Disclosure statement. This is published with the article. It must therefore be completed in full sentences and contain the exact wording you wish to be published.
   a. State the initials, alongside each funding source, of each author to receive each grant. For example: ""This work was supported by the National Institutes of

Health (####### to AM; ###### to CJ) and the National Science Foundation (###### to AM).""

Response: Please see the revised financial disclosure statement. “This project has been funded by the United Nations Development Program - United Nations Population Fund– World Health Organization – World Bank Special Program of Research in Human Reproduction (HRP), to PA (WHO PO # 202833289). In addition, the project received support from the National Cancer Institute, National Institutes of Health, under Contract No. 75N91019D00024. The content of this publication does not necessarily reflect the views or policies of the Department of Health and Human Services, nor does mention of trade names, commercial products, or organizations imply endorsement by the U.S. Government.”

b. State what role the funders took in the study. If the funders had no role in your study, please state: “The funders had no role in study design, data collection and analysis, decision to publish, or preparation of the manuscript.”

Response: The funders reviewed the project proposal and provided suggestions for the interview guide. The funders also helped in the recruitment of the study participants by providing an initial introduction to the research team. Once this connection was made, the funders were not involved in the data collection, management, and the analyses of the data. In addition, they had no role in the decision to publish or preparation of the manuscript.

Sincerely,
Prajakta Adsul

Response for Editorial comments dated 11 May, 2024

Dear Dr. Shrestha and Reviewers,

We would like to express our gratitude to you for taking the time to review our manuscript entitled, “Assessing the Global Implications of the COVID-19 Pandemic on Cervical Cancer Screening Initiatives: A Comprehensive Evaluation” and inviting us to submit a revised draft. Thank you for providing insightful feedback and constructive comments. Thus, it is with great pleasure that we resubmit our article for further consideration. We have incorporated changes that reflect your suggestions and inquiries. We hope that our edits and responses we provide below satisfactorily address all the issues and concerns you and the reviewers have noted.

To facilitate your review of our revisions, the following is a point-by-point response to the questions and comments delivered in your letter dated 11 May, 2024.

Editor Comments:

1. Please review your reference list to ensure that it is complete and correct. If you have cited papers that have been retracted, please include the rationale for doing so in the manuscript text, or remove these references and replace them with relevant current references. Any changes to the reference list should be mentioned in the rebuttal letter

that accompanies your revised manuscript. If you need to cite a retracted article, indicate the article's retracted status in the References list and also include a citation and full reference for the retraction notice.

- Response: Thank you for the valuable comment. All references have been checked for completeness and correctness and none have been retracted as of 1 June, 2024.

2. Please provide additional details regarding participant consent. In the ethics statement in the Methods and online submission information, please ensure that you have specified (1) whether consent was informed and (2) what type you obtained (for instance, written or verbal, and if verbal, how it was documented and witnessed)."
    - Response: Thank you for clarifying details of participant consent. The following has been added to the Methods section – "Written consent was obtained when the interview was scheduled. If, for some reason, the participant did not have time to complete the written consent, verbal consent was obtained with the interviewer as the witness on the recorded Zoom calls." (Lines 71-74).

3. Please include a complete copy of PLOS' questionnaire on inclusivity in global research in your revised manuscript. Our policy for research in this area aims to improve transparency in the reporting of research performed outside of researchers' own country or community. The policy applies to researchers who have travelled to a different country to conduct research, research with Indigenous populations or their lands, and research on cultural artefacts. The questionnaire can also be requested at the journal's discretion for any other submissions, even if these conditions are not met. Please find more information on the policy and a link to download a blank copy of the questionnaire here: https://journals.plos.org/globalpublichealth/s/best-practices-in-research-reporting. Please upload a completed version of your questionnaire as Supporting Information when you resubmit your manuscript.
    - Response: Thank you for alerting us to this questionnaire on inclusivity in global research. A completed copy has been provided with the re-submission.

Reviewer #1

1. Thank you for the opportunity to review! This paper presents important findings on how healthcare systems worldwide responded to the COVID-19 pandemic, illustrating how healthcare systems in LMICs faced different challenges from those in HIC. These results will be a valuable contribution to the literature on emergency preparedness, specifically relating to challenges in managing chronic diseases in low-resource settings.
    - Response: Thank you very much for your positive feedback on our manuscript.

Reviewer #2

1. Interesting and timely manuscript resulting in first-hand accounts from around the globe of how the pandemic impacted cervical cancer prevention services. It is nice to see that the authors present opportunities as well as challenges that may help guide future research. Detailed comments below.
    - Response: Thank you for taking the time to provide detailed comments. Responses and feedback provided below.

2. Abstract: "supporting countries' abilities to mobilize resources."

- Response: Thank you for this revision. This change has been reflected in the abstract.

3. In the methods, it would be useful to have some details about the simultaneous translation (in person? AI?) and/or number of languages the interviews took place in (X in English, X in other languages?)
    - Response: You raise an important question. The following has been added into the Methods sections – “Simultaneous translation, which consisted of two live interpreters; one on an English channel with the interviewers and one on a French or Spanish channel with the participant, was provided for non-English speakers. There were a total of three interviews which required these efforts (two in French and one in Spanish).” (Lines 86-89).

4. In methods, although the participant sample is described, additional details that speak to sample representatives should be included. It’s a qualitative study, but it’s still helpful to know how many participants of how many categories/per country were included.
    - Response: You make a fair assessment. To give representation from each WHO region, we have provided demographic information on participants from each region. Giving individual country names together with participant profession may disclose participant identity. We have added additional details on participant representation in the next question.

5. Also methods: The sample of countries requires more description at least in terms of income (for example, X low income countries, X low-middle, X upper middle, etc.). Would be useful to know how many countries were included and which ones/how many of those have existing HPV screening and/or vaccination programs (as opposed to pilot or research projects) and since when (or at least giving a range of years or older vs. new?). This is important contextual information that should be discussed within each analysis section as we can expect the impact of the pandemic to be different on established programs vs. research/pilot projects and income level of each country. For example, some themes may be more relevant to specific countries/regions with more resources (like telemedicine) and that should be acknowledged.
    - Response. Thank you for this suggestion. The following have been added to Results section to reflect more details on country income status and existing HPV screening programs - “Out of the 25 countries, in accordance with the World Bank, two is designated as low-income, 10 as lower-middle income, 11 as higher-middle income, and two as high-income [23]. 87% of included countries have a national cervical cancer screening program and approximately half have existing HPV testing programs, introduced from 2011-2023 [24]” (Lines 115-118).

6. In general, conclusions are over-drawn. Given the methods and sample size and selection (overall and per country) results can be categorized as exploratory at best. For example, in Lines 200-216, one person’s opinion does not mean that is actually the case. Interpretation should be revised from “improvement in awareness among the populations was an important advantage” to “may be a potential advantage”. This is the case throughout the manuscript.
    - Response: Thank you for bringing this to our attention. Interpretations have been revised in lines 234-235, 356, and 358 to reflect less certainty in conclusions.

7. In Results, it would be important to know which themes were most/least common and which were most/least often associated with particular regions/countries (maybe a table?). It would also be good to know if different themes were most often mentioned or were considered more important by different types of interviewees (ministry officials, providers, program managers, etc.).
    - Response: Thank you for this suggestion. Quantifying the data points as suggested to explore associations with country characteristics might dilute the rich contextual information provided through the open-ended qualitative methods of inquiry used in this study. Furthermore, there is a potential to misinterpret these associations at a country-level when the interviewee may be referring to associations at the project level. Certainly, in future research informed by this qualitative inquiry, we plan to use appropriate quantitative methods to continue to build evidence for implementation.

8. Line 143-146: discusses VIA as the screening method, not HPV testing, which is confusing given the abstract which specifically refers to HPV testing. Clarifying screening methods used in the different countries would be helpful, as these two methods are expected to present at least some different challenges as a result of COVID.
    - Response: Thank you for highlighting a bit of confusion – you are correct in that some countries are still using VIA as standard-of-care. Clarification has been added into Line 155 "A tertiary-level health professional from the SEAR region, not yet having implemented HPV screening, noted the impacts of COVID-19 on the workforce for VIA:"

9. Line 148-152: same issue as above, would be helpful to clarify the context in which this was noted (a country with an existing HPV program but no self-sampling? Introduction of HPV as a result of the pandemic?)
    - Response: Thank you for bringing this to our attention. Clarification has been added into Line 163 – "While HPV testing was still being rolled out in some regions, participants noted that the pandemic resulted in a clear need to collect cervical samples with a limited number of healthcare workers, leading to favoring of HPV DNA tests which allowed self-sampling."

10. Line 167-168: "the poorly-funded programs". This also assumes that all countries involved were in low-resource-settings, but that is not clear from the methods which discusses a range of global regions. In any case, LMICs will vary greatly in available resources and some may have much funded programs than others.
    - Response: Thank you for this inquiry. As described above, further details on country income status have been added to the Methods section. We have re-envisioned the choice of the word "poorly-funded" and changed the language to underfunded. In general, public health and cancer prevention programs are underfunded, even in high-income countries – we intended to express even further diversion of resources. Revisions have been included in Lines 183-184 - "During the pandemic, participants stressed that any focus governmental officials had on implementing CC screening programs, as well as human resources, were diverted to COVID-19 testing and care, which significantly impeded the already underfunded public health and cancer prevention programs."

11. Line 234-235: incidence already slowed down

- Response: Thank you for catching that. Sentence has been rephrased as such – "These advancements in testing capabilities can be leveraged for HPV testing, especially as the incidence of COVID-19 has slowed down" (Lines 254-255).

12. Line 259-260: Not clear why fragmented healthcare systems are crucial for screening?
    - Response: You raise an important question in the incorrect wording of this sentence. Please see revised sentence in Lines 281-283 – "Some participants shared concerns about the impact of the pandemic on the fragmented healthcare systems, while adding that well-functioning healthcare systems are crucial for screening programs to remain successful."

13. Line 320-321: it's curious that COVID misinformation and vaccine hesitancy were not mentioned in the interviews, since that has been an often-reported outcome of the pandemic in various countries including LMICs. Perhaps those folks have less contact with the health system and that skews the perception. But the literature on these issues should be acknowledged.
    - Response: Thank you for your valuable insights. The main purpose of the interviews was assessing implementation strategies surrounding HPV testing programs. We recognized that since we were conducting the interviews in the midst of the pandemic, we wanted to ask: "How has COVID affected the current implementation efforts?" We can confirm that the topics of misinformation and vaccine hesitancy were not brought up in the interviews, perhaps due to the brevity of the interview section hence adding literature on these topics would not support and would be misrepresenting the conversations we had.

14. Lines 328-339: Again, some caution in interpreting results from these interviews is needed here, firm conclusions cannot be drawn from the data.
    - Response: Thank you for highlighting this. As mentioned above, interpretation has been revised as the following – "While many health systems revealed poor coordination efforts, there was a need to shift the ways in which some practices were conducted. The rise in virtual activities associated with social distancing protocols may have resulted in increased acceptance and usage of telemedicine and web-delivered trainings for the healthcare professional workforce. HPV DNA tests, in which samples can be collected without the need of a clinician, seemed to gain increased acceptance among healthcare professionals and public health decision-makers as a method to continue screening in times of health emergencies, despite the cost." (Lines 354-360).

15. Remove commas in Lines 45, 50, 93, 175, 275, 301, 338
    - Response: Thank you for the suggestions. Commas have been removed from Lines 45, 50, 93 (now 101), 175 (now 193), 275 (now 299), 301 (now 326), and 338 (now 365).

Additional comments from the Editor

Thank you for detailing further requirements for submission of our manuscript entitled "Assessing the Global Implications of the COVID-19 Pandemic on Cervical Cancer Screening Initiatives: A Comprehensive Evaluation". Please see the below response to your inquiries.

5. In the online submission form, you indicated that [Insert text from online submission form here].

   All PLOS journals now require all data underlying the findings described in their manuscript to be freely available to other researchers, either
   1. In a public repository,
   2. Within the manuscript itself, or
   3. Uploaded as supplementary information.

   This policy applies to all data except where public deposition would breach compliance with the protocol approved by your research ethics board. If your data cannot be made publicly available for ethical or legal reasons (e.g., public availability would compromise patient privacy), please explain your reasons by return email and your exemption request will be escalated to the editor for approval. Your exemption request will be handled independently and will not hold up the peer review process, but will need to be resolved should your manuscript be accepted for publication. One of the Editorial team will then be in touch if there are any issues."
   - Response: Thank you for bringing this to our attention. All data pertinent to the question being answered in this paper are included within the manuscript itself. Additional contextual information can be obtained by contacting the corresponding author.

6. Please remove the old version of the manuscript file and retain only the revised version.
   - Response: In the re-submission, we have included a revised version of the manuscript including track changes as well as a version with the changes accepted.

7. Please send a completed 'Competing Interests' statement, including any COIs declared by your co-authors. If you have no competing interests to declare, please state "The authors have declared that no competing interests exist".
   - Response: The authors have declared that no competing interests exist.

8. Please amend your detailed Financial Disclosure statement. This is published with the article. It must therefore be completed in full sentences and contain the exact wording you wish to be published.
   c. State the initials, alongside each funding source, of each author to receive each grant. For example: ""This work was supported by the National Institutes of Health (####### to AM; ###### to CJ) and the National Science Foundation (###### to AM).""
      - Response: Please see the revised financial disclosure statement.
        "This project has been funded by the United Nations Development Program - United Nations Population Fund– World Health Organization – World Bank Special Program of Research in Human Reproduction (HRP), to PA (WHO PO # 202833289). In addition, the project received support from the National Cancer Institute, National Institutes of Health, under Contract No. 75N91019D00024. The content of this publication does not

necessarily reflect the views or policies of the Department of Health and Human Services, nor does mention of trade names, commercial products, or organizations imply endorsement by the U.S. Government."

d. State what role the funders took in the study. If the funders had no role in your study, please state: "The funders had no role in study design, data collection and analysis, decision to publish, or preparation of the manuscript."
    - Response: The funders reviewed the project proposal and provided suggestions for the interview guide. The funders also helped in the recruitment of the study participants by providing an initial introduction to the research team. Once this connection was made, the funders had no involvement in the data collection, management, and the analyses of the data. In addition, they had no role in the decision to publish or preparation of the manuscript.

Again, thank you for giving us the opportunity to strengthen our manuscript with your insightful comments and queries. We have worked diligently to incorporate your feedback and hope these revisions persuade you to accept our submission.

Sincerely,
Prajakta Adsul
