# Supplementary material for: Assessing the global implications of the COVID-19 pandemic on the cervical cancer elimination initiative
Source: PLOS Glob Public Health. 2025 Apr 28;5(4):e0004419. doi: 10.1371/journal.pgph.0004419 (PMC12036855; doi:10.1371/journal.pgph.0004419)
Supplement: Attachment — Submitted filename: LOS_Adsul_UNM-CADIS_2025.2_PLOS global rnr.pdf [file pgph.0004419.s006.pdf]

COMPREHENSIVE CANCER CENTER

HEALTH SCIENCES
CENTER FOR ADVANCING DISSEMINATION & IMPLEMENTATION SCIENCE

**February 22, 2025**

Dear Editors and Reviewers,

We sincerely appreciate your time and effort in reviewing our manuscript "Assessing the Global Implications of the COVID-19 Pandemic on Cervical Cancer Screening Initiatives" and for inviting us to submit a revised version. We are pleased to resubmit our manuscript for further consideration, having incorporated revisions that align with your suggestions and inquiries.

To facilitate your review of our revisions, the following is a point-by-point response to the questions and comments delivered in your letter dated 24 January, 2025.

**Reviewer #2:**

1. This is a timely and important manuscript that highlights challenges, but more significantly, opportunities to expand and scale-up cervical cancer prevention efforts by leveraging changes stemming from the COVID pandemic. The sample is diverse, the analysis is rigorous, and the conclusions are well-connected to the data. There are a few areas where clearer writing or adding an explanatory sentence would be beneficial. I also appreciate the desire to connect the manuscript to the implementation literature, but discussing the framework used in the parent project is confusing (I was expecting to see more of it throughout the results/conclusions) and not immediately relevant to these findings. Specific comments are on the margins of the PDF.

   **Response:** Thank you for your feedback on our manuscript. We agree with that the framework is not relevant to the current findings and have removed it from the manuscript.

2. Comment on line 30: CC-related? or all other non-emergency healthcare services?

   **Response:** Thank you for this inquiry. Both references cited are related to cancer care services. Thus, we have revised the sentence to read:
   *"Additionally, community members lost trust in the healthcare system, <u>in particular as it relates to cancer care</u>".*

3. Comment on line 48: this sentence is long and a bit confusing - does it matter here that the larger study is guided by EPIS (since it is not further described)? Was EPIS considered for this study too? A bit unclear.

   **Response:** Thank you. As noted earlier, this sentence has been revised to simplify and eliminate the mention of the EPIS framework
   *"The study is a part of a larger initiative aimed at generating practice-based evidence for strategies and approaches that support the implementation of ongoing HPV DNA-based screening and treatment programs".*

Department of Epidemiology, Biostatistics & Preventive Medicine | Phone: 505-272-7351 | Email: padsul@salud.unm.edu
Mailing Address: MSC07-4025, Rm G-11 University of New Mexico | MSC10 5550 | Albuquerque, NM 87131
**hsc.unm.edu/research/centers-programs/cadis/**

4. Comment on line 77: So this is the EPIS part? Would be helpful to explain where the Covid questions fit i or if they were added post-hoc given the circumstances

   **Response:** Thank you for this comment. We have removed the reference to EPIS as noted earlier.

5. Comment on line 99: So two people coded and a third was assigned to adjudicate? Unclear

   **Response:** We have further clarified this sentence to read: During initial coding, two team members independently coded select transcripts, and a third coder helped resolve discrepancies through discussion and consensus.

6. Comment on line 113: "is designated" to be edited to "were designated"

   **Response:** Thank you for bringing this to our attention. The sentence has been revised accordingly.

7. Comment on line 114 on cervical cancer: CC for consistency

   **Response**: We have revised accordingly.

8. Comment on line 116 on participant professions: Participants' roles

   **Response**: Thank you for this comment. We are describing the general occupations that the interview participants held during the time of interview. 'Participant role' does not fully address this point.

9. Comment on line 126: would be helpful to explain why or if these programs were concentrated in specific regions?

   **Response:** Thank you for this insightful question. We have included a couple reasons that participants gave us. The sentence has been revised to read:
   ***"****Interestingly, there were some countries where programs continued uninterrupted by COVID-19 for reasons such as the essential nature of CC screening and the ability to leverage COVID-19 testing sites for HPV."*

10. Comment on line 369 on "others that maybe reflective...": and this may be

    **Response:** Thank you for this edit. The sentence has been revised accordingly.

11. Comment on line 379: "rates" is an odd word to choose, this is not just about rates. Maybe issues? Services?

    **Response:** We have revised the sentence to reference issues and services: Further, the primary focus of our study was on screening services so when participants did comment on issues regarding vaccination,

precancer treatment, and cancer treatment, we did not probe to learn more about the impact of COVID-19 on those services.

**Other comments found in the attached Word document:**

12. Comment in line 5 on the treatment of positives: More appropriate to say "treatment of those with cervical cancer or related malignancies' treatment is not based on HPV positivity but on presence of cervical disease or precancerous lesions

    **Response:** Thank you for this suggestion. We have made this edit in the revised manuscript: However, it is preventable through interventions such as human papillomavirus (HPV) vaccination and regular CC screening followed by treatment of those with CC or related malignancies.

13. Comment in line 60 on the Methods section: There is a missing part about what type of design is the research approach.

    **Response:** Thank you for your valuable input. We have now added a sentence to the methods section. "This study employs a qualitative research approach using in-depth interviews with program implementers to explore the implementation of cervical cancer screening programs globally [21]."

14. Comment in line 87 on the Dedoose qualitative software: Reference to software, usually Company and city and version of software

    **Response:** Thank you for highlighting this. The manuscript has now been revised to denote Los Angeles, CA and Version 9.2.22.

**Reviewer #3:**

1. The introduction provides a clear context about the pandemic's impact on cervical cancer screening but could better emphasize the novelty of this study compared to existing research. Highlight specific gaps in the current literature that this study aims to address.
   Recommendations: Add a paragraph summarizing how the study uniquely contributes to understanding the global implications of COVID-19 on cervical cancer screening.

   **Response:** Thank you for this comment. We believer that lines 24-47 in the introduction provide the analysis of the current literature that collectively provide the rationale for the current study. In order to draw attention to this for the reader, we have added a sentence at the end of that section,

   *"These challenges and potential leverage points for supporting implementation of preventive services have not been systematically studied in the context of cervical cancer screening programs across the globe."*

2. While six themes are identified, some sections, such as Theme 2 (Fear of contracting COVID-19) and Theme 5 (Fragmentation of healthcare systems), need more robust linking to the findings' broader

implications. Recommendations: Expand on how these themes can guide policymakers in strengthening healthcare systems for future resilience.

**Response:** Thank you for raising this important point. In responding to the next questions, we have revised and strengthened the discussion to expand on the themes.

3. The manuscript describes using a grounded theory approach but does not detail how inter-coder reliability was ensured or how saturation was determined. Recommendations: Include a brief explanation of inter-coder reliability measures and the method used to determine thematic saturation.

   **Response:** We appreciate your thoughtful comment. In regard to inter-coder reliability and to address the comments of Reviewer #2, we have revised the manuscript to read:
   *"During initial coding, two team members independently coded select transcripts, and a third coder helped to resolve discrepancies through discussion and consensus."*

   In regard to thematic saturation, we used the notes that were taken and discussed after each interview common points interviewees were mentioning. We determined saturation when we began to hear the same points from interviewees of different settings. We have added additional clarity to a sentence in the Methods section to read:
   *"We achieved data saturation at 32 interviews, determined through discussions between study team members after each interview on common points we kept on hearing, even from interviewees of different settings."*

4. The limitations section is thorough but lacks emphasis on potential biases due to the reliance on WHO-selected participants. Recommendations: Explicitly mention how participant selection might skew results and suggest strategies to mitigate this in future research.

   **Response:** Thank you for bringing this to our attention. We have added these sentences into the limitations section:
   *"Moreover, the method of participant selection may have introduced bias, as perspectives and experiences may not be representative of a broader population of CC experts. Future research may benefit from employing a randomized selection process to minimize such biases."*

5. The discussion section could better integrate the findings with actionable recommendations for programmatic improvements post-pandemic. Recommendations: Propose specific strategies for leveraging pandemic-induced infrastructure improvements (e.g., molecular testing platforms for HPV screening) in LMICs.

   **Response:** Thank you for this suggestion. We have added the following paragraph to the discussion section to address this:
   *"These shifts in healthcare infrastructure and service delivery, such as the increased capacity for HPV testing and the growing acceptance of self-sampling, offer an important opportunity to enhance CC*

Department of Epidemiology, Biostatistics & Preventive Medicine | Phone: 505-272-7351 | Email: padsul@salud.unm.edu
Mailing Address: MSC07-4025, Rm G-11 University of New Mexico | MSC10 5550 | Albuquerque, NM 87131
**hsc.unm.edu/research/centers-programs/cadis/**

*prevention programs moving forward. For instance, the widespread use of PCR machines during the pandemic can be leveraged for HPV testing, providing a cost-effective and scalable solution for CC screening in LMICs. Additionally, the training of technicians in PCR testing can be adapted to include HPV testing, expanding the workforce's ability to support CC screening and prevention efforts. Similarly, telemedicine [25] and self-sampling [26], which gained momentum during the pandemic, can play a significant role in increasing access to CC prevention services. Offering HPV self-sampling kits and introducing virtual care could reduce barriers to access. Expanding the use of these approaches, even post-pandemic, would ensure that CC screening methods remain accessible to underserved populations."*

Again, thank you for giving us the opportunity to strengthen our manuscript with your insightful comments and queries. Please do not hesitate to email me (padsul@salud.unm.edu), should you have any questions regarding the information provided in this letter.

Sincerely,

**Prajakta Adsul, MBBS, MPH, PhD**

Inaugural Director, Center for Advancing Dissemination and Implementation Science, University of New Mexico Health Sciences Center
Assistant Professor, Division of Epidemiology, Biostatistics, and Preventive Medicine, Department of Internal Medicine, School of Medicine, University of New Mexico
Member, Cancer Control and Population Sciences Research Program, Comprehensive Cancer Center, University of New Mexico
