# Supplementary material for: Assessing the global implications of the COVID-19 pandemic on the cervical cancer elimination initiative
Source: PLOS Glob Public Health. 2025 Apr 28;5(4):e0004419. doi: 10.1371/journal.pgph.0004419 (PMC12036855; doi:10.1371/journal.pgph.0004419)
Supplement: Attachment — Submitted filename: Response to Reviewers_2024_7.30.docx [file pgph.0004419.s005.docx]

**Response for technical comments dated Jul 1, 2024**

1. Thank you for providing the requested questionnaire for global inclusivity. However, we note that the questionnaire has been submitted as an ""other"" file type. Kindly resubmit the global inclusivity questionnaire as Supporting file

**Response**: We have now uploaded the inclusivity questionnaire as a Supporting file.

2. PLOS journals require authors to make all data necessary to replicate their study’s findings publicly available without restriction at the time of publication. When specific legal or ethical restrictions prohibit public sharing of a data set, authors must indicate how others may obtain access to the data.

In line with our goal of ensuring long-term data availability to all interested researchers, PLOS’ Data Policy states that authors cannot be the sole named individuals responsible for ensuring data access (http://journals.plos.org/plosone/s/data-availability#loc-acceptable-data-sharing-methods). As you have noted, however, your institution does not currently have a point of contact for fielding requests for sensitive data. In light of this, please consider whether any of the following would be possible:

a) Can you make a de-identified, anonymized, or aggregated data set publicly available in a repository or, if that is not possible, in your manuscript’s Supporting Information files? For a list of recommended repositories, some of which are able to hold sensitive data, see here: https://journals.plos.org/plosone/s/recommended-repositories

**Response:** Please note that in previous responses we have clarified that all data from the study are being included in the manuscript itself. There is no additional data related to this manuscript and we are not requesting any exemptions in this regard. We have updated the online submission form accordingly.

b) Are there any institutional representatives who did not collaborate in the study and are not listed as authors on the manuscript who would be able to hold the data and respond to external requests for data access? If so, please provide their contact information (i.e., email address). Please also provide details on how you will ensure persistent or long-term data storage and availability. For instance, data might be stored in two independent locations.

**Response:** Since we are not requesting any exemptions in this regard and are not storing any additional data in the repository, we do not believe an institutional representative is needed to provide access to the data. All data for this study are included within the manuscript. We have updated the online submission form accordingly.

**Response for technical comments dated 9 June, 2024**

Dear Editor,

Thank you for detailing further requirements for submission of our manuscript entitled “Assessing the Global Implications of the COVID-19 Pandemic on Cervical Cancer Screening Initiatives: A Comprehensive Evaluation”. Please see the below response to your inquiries.

1. In the online submission form, you indicated that [Insert text from online submission form here].

All PLOS journals now require all data underlying the findings described in their manuscript to be freely available to other researchers, either

1. In a public repository,

2. Within the manuscript itself, or

3. Uploaded as supplementary information.

This policy applies to all data except where public deposition would breach compliance with the protocol approved by your research ethics board. If your data cannot be made publicly available for ethical or legal reasons (e.g., public availability would compromise patient privacy), please explain your reasons by return email and your exemption request will be escalated to the editor for approval. Your exemption request will be handled independently and will not hold up the peer review process, but will need to be resolved should your manuscript be accepted for publication. One of the Editorial team will then be in touch if there are any issues."

**Response:** Thank you for bringing this to our attention. All data pertinent to the question being answered in this paper are included within the manuscript itself. Additional contextual information can be obtained by contacting the corresponding author.

2. Please remove the old version of the manuscript file and retain only the revised version.

**Response:** In the re-submission, we have included a revised version of the manuscript including track changes as well as a version with the changes accepted.

3. Please send a completed 'Competing Interests' statement, including any COIs declared by your co-authors. If you have no competing interests to declare, please state "The authors have declared that no competing interests exist".

**Response:** The authors have declared that no competing interests exist.

4. Please amend your detailed Financial Disclosure statement. This is published with the article. It must therefore be completed in full sentences and contain the exact wording you wish to be published.
   a. State the initials, alongside each funding source, of each author to receive each grant. For example: ""This work was supported by the National Institutes of Health (####### to AM; ###### to CJ) and the National Science Foundation (###### to AM).""

**Response**: Please see the revised financial disclosure statement.

“This project has been funded by the United Nations Development Program - United Nations Population Fund– World Health Organization –World Bank Special Program of Research in Human Reproduction (HRP), to PA (WHO PO # 202833289). In addition, the project received support from the National Cancer Institute, National Institutes of Health, under Contract No. 75N91019D00024. The content of this publication does not necessarily reflect the views or policies of the Department of Health and Human Services, nor does mention of trade names, commercial products, or organizations imply endorsement by the U.S. Government.”

b. State what role the funders took in the study. If the funders had no role in your study, please state: “The funders had no role in study design, data collection and analysis, decision to publish, or preparation of the manuscript.”

**Response:** The funders reviewed the project proposal and provided suggestions for the interview guide. The funders also helped in the recruitment of the study participants by providing an initial introduction to the research team. Once this connection was made, the funders were not involved in the data collection, management, and the analyses of the data. In addition, they had no role in the decision to publish or preparation of the manuscript.

Sincerely,

Prajakta Adsul

**Response for Editorial comments dated 11 May, 2024**

Dear Dr. Shrestha and Reviewers,

We would like to express our gratitude to you for taking the time to review our manuscript entitled, “Assessing the Global Implications of the COVID-19 Pandemic on Cervical Cancer Screening Initiatives: A Comprehensive Evaluation” and inviting us to submit a revised draft. Thank you for providing insightful feedback and constructive comments. Thus, it is with great pleasure that we resubmit our article for further consideration. We have incorporated changes that reflect your suggestions and inquiries. We hope that our edits and responses we provide below satisfactorily address all the issues and concerns you and the reviewers have noted.

To facilitate your review of our revisions, the following is a point-by-point response to the questions and comments delivered in your letter dated 11 May, 2024.

**Editor Comments:**

1. Please review your reference list to ensure that it is complete and correct. If you have cited papers that have been retracted, please include the rationale for doing so in the manuscript text, or remove these references and replace them with relevant current references. Any changes to the reference list should be mentioned in the rebuttal letter that accompanies your revised manuscript. If you need to cite a retracted article, indicate the article’s retracted status in the References list and also include a citation and full reference for the retraction notice.
   - Response: Thank you for the valuable comment. All references have been checked for completeness and correctness and none have been retracted as of 1 June, 2024.
2. Please provide additional details regarding participant consent. In the ethics statement in the Methods and online submission information, please ensure that you have specified (1) whether consent was informed and (2) what type you obtained (for instance, written or verbal, and if verbal, how it was documented and witnessed).”
   - Response: Thank you for clarifying details of participant consent. The following has been added to the Methods section – “Written consent was obtained when the interview was scheduled. If, for some reason, the participant did not have time to complete the written consent, verbal consent was obtained with the interviewer as the witness on the recorded Zoom calls.” (Lines 71-74).
3. Please include a complete copy of PLOS’ questionnaire on inclusivity in global research in your revised manuscript. Our policy for research in this area aims to improve transparency in the reporting of research performed outside of researchers’ own country or community. The policy applies to researchers who have travelled to a different country to conduct research, research with Indigenous populations or their lands, and research on cultural artefacts. The questionnaire can also be requested at the journal’s discretion for any other submissions, even if these conditions are not met. Please find more information on the policy and a link to download a blank copy of the questionnaire here: [https://journals.plos.org/globalpublichealth/s/best-practices-in-research-reporting](https://urldefense.com/v3/__https:/journals.plos.org/globalpublichealth/s/best-practices-in-research-reporting__;!!K-Hz7m0Vt54!hSYcJ2loF-SjdgZODDueQVN58gYJZDpQ45_TNROQdOdUdNcS5rElt4oBvujtTdMn7p-NmdkXKWt6rxng$). Please upload a completed version of your questionnaire as Supporting Information when you resubmit your manuscript.
   - Response: Thank you for alerting us to this questionnaire on inclusivity in global research. A completed copy has been provided with the re-submission.

**Reviewer #1**

1. Thank you for the opportunity to review! This paper presents important findings on how healthcare systems worldwide responded to the COVID-19 pandemic, illustrating how healthcare systems in LMICs faced different challenges from those in HIC. These results will be a valuable contribution to the literature on emergency preparedness, specifically relating to challenges in managing chronic diseases in low-resource settings.
   - Response: Thank you very much for your positive feedback on our manuscript.

**Reviewer #2**

1. Interesting and timely manuscript resulting in first-hand accounts from around the globe of how the pandemic impacted cervical cancer prevention services. It is nice to see that the authors present opportunities as well as challenges that may help guide future research. Detailed comments below.
   - Response: Thank you for taking the time to provide detailed comments. Responses and feedback provided below.
2. Abstract: “supporting countries’ abilities to mobilize resources.”
   - Response: Thank you for this revision. This change has been reflected in the abstract.
3. In the methods, it would be useful to have some details about the simultaneous translation (in person? AI?) and/or number of languages the interviews took place in (X in English, X in other languages?)
   - Response: You raise an important question. The following has been added into the Methods sections – “Simultaneous translation, which consisted of two live interpreters; one on an English channel with the interviewers and one on a French or Spanish channel with the participant, was provided for non-English speakers. There were a total of three interviews which required these efforts (two in French and one in Spanish).” (Lines 86-89).
4. In methods, although the participant sample is described, additional details that speak to sample representatives should be included. It’s a qualitative study, but it’s still helpful to know how many participants of how many categories/per country were included.
   - Response: You make a fair assessment. To give representation from each WHO region, we have provided demographic information on participants from each region. Giving individual country names together with participant profession may disclose participant identity. We have added additional details on participant representation in the next question.
5. Also methods: The sample of countries requires more description at least in terms of income (for example, X low income countries, X low-middle, X upper middle, etc.). Would be useful to know how many countries were included and which ones/how many of those have existing HPV screening and/or vaccination programs (as opposed to pilot or research projects) and since when (or at least giving a range of years or older vs. new?). This is important contextual information that should be discussed within each analysis section as we can expect the impact of the pandemic to be different on established programs vs. research/pilot projects and income level of each country. For example, some themes may be more relevant to specific countries/regions with more resources (like telemedicine) and that should be acknowledged.
   - Response. Thank you for this suggestion. The following have been added to Results section to reflect more details on country income status and existing HPV screening programs - “Out of the 25 countries, in accordance with the World Bank, two is designated as low-income, 10 as lower-middle income, 11 as higher-middle income, and two as high-income [23]. 87% of included countries have a national cervical cancer screening program and approximately half have existing HPV testing programs, introduced from 2011-2023 [24]” (Lines 115-118).
6. In general, conclusions are over-drawn. Given the methods and sample size and selection (overall and per country) results can be categorized as exploratory at best. For example, in Lines 200-216, one person’s opinion does not mean that is actually the case. Interpretation should be revised from “improvement in awareness among the populations was an important advantage” to “may be a potential advantage”. This is the case throughout the manuscript.
   - Response: Thank you for bringing this to our attention. Interpretations have been revised in lines 234-235, 356, and 358 to reflect less certainty in conclusions.
7. In Results, it would be important to know which themes were most/least common and which were most/least often associated with particular regions/countries (maybe a table?). It would also be good to know if different themes were most often mentioned or were considered more important by different types of interviewees (ministry officials, providers, program managers, etc.).
   - Response: Thank you for this suggestion. Quantifying the data points as suggested to explore associations with country characteristics might dilute the rich contextual information provided through the open-ended qualitative methods of inquiry used in this study. Furthermore, there is a potential to misinterpret these associations at a country-level when the interviewee may be referring to associations at the project level. Certainly, in future research informed by this qualitative inquiry, we plan to use appropriate quantitative methods to continue to build evidence for implementation.
8. Line 143-146: discusses VIA as the screening method, not HPV testing, which is confusing given the abstract which specifically refers to HPV testing. Clarifying screening methods used in the different countries would be helpful, as these two methods are expected to present at least some different challenges as a result of COVID.
   - Response: Thank you for highlighting a bit of confusion – you are correct in that some countries are still using VIA as standard-of-care. Clarification has been added into Line 155 “A tertiary-level health professional from the SEAR region, not yet having implemented HPV screening, noted the impacts of COVID-19 on the workforce for VIA:”
9. Line 148-152: same issue as above, would be helpful to clarify the context in which this was noted (a country with an existing HPV program but no self-sampling? Introduction of HPV as a result of the pandemic?)
   - Response: Thank you for bringing this to our attention. Clarification has been added into Line 163 – “While HPV testing was still being rolled out in some regions, participants noted that the pandemic resulted in a clear need to collect cervical samples with a limited number of healthcare workers, leading to favoring of HPV DNA tests which allowed self-sampling.”
10. Line 167-168: “the poorly-funded programs”. This also assumes that all countries involved were in low-resource-settings, but that is not clear from the methods which discusses a range of global regions. In any case, LMICs will vary greatly in available resources and some may have much funded programs than others.
    - Response: Thank you for this inquiry. As described above, further details on country income status have been added to the Methods section. We have re-envisioned the choice of the word “poorly-funded” and changed the language to underfunded. In general, public health and cancer prevention programs are underfunded, even in high-income countries – we intended to express even further diversion of resources. Revisions have been included in Lines 183-184 - “During the pandemic, participants stressed that any focus governmental officials had on implementing CC screening programs, as well as human resources, were diverted to COVID-19 testing and care, which significantly impeded the already underfunded public health and cancer prevention programs.”
11. Line 234-235: incidence already slowed down
    - Response: Thank you for catching that. Sentence has been rephrased as such – “These advancements in testing capabilities can be leveraged for HPV testing, especially as the incidence of COVID-19 has slowed down” (Lines 254-255).
12. Line 259-260: Not clear why fragmented healthcare systems are crucial for screening?
    - Response: You raise an important question in the incorrect wording of this sentence. Please see revised sentence in Lines 281-283 – “Some participants shared concerns about the impact of the pandemic on the fragmented healthcare systems, while adding that well-functioning healthcare systems are crucial for screening programs to remain successful.”
13. Line 320-321: it’s curious that COVID misinformation and vaccine hesitancy were not mentioned in the interviews, since that has been an often-reported outcome of the pandemic in various countries including LMICs. Perhaps those folks have less contact with the health system and that skews the perception. But the literature on these issues should be acknowledged.
    - Response: Thank you for your valuable insights. The main purpose of the interviews was assessing implementation strategies surrounding HPV testing programs. We recognized that since we were conducting the interviews in the midst of the pandemic, we wanted to ask: “How has COVID affected the current implementation efforts?” We can confirm that the topics of misinformation and vaccine hesitancy were not brought up in the interviews, perhaps due to the brevity of the interview section hence adding literature on these topics would not support and would be misrepresenting the conversations we had.
14. Lines 328-339: Again, some caution in interpreting results from these interviews is needed here, firm conclusions cannot be drawn from the data.
    - Response: Thank you for highlighting this. As mentioned above, interpretation has been revised as the following – “While many health systems revealed poor coordination efforts, there was a need to shift the ways in which some practices were conducted. The rise in virtual activities associated with social distancing protocols may have resulted in increased acceptance and usage of telemedicine and web-delivered trainings for the healthcare professional workforce. HPV DNA tests, in which samples can be collected without the need of a clinician, seemed to gain increased acceptance among healthcare professionals and public health decision-makers as a method to continue screening in times of health emergencies, despite the cost.” (Lines 354-360).
15. Remove commas in Lines 45, 50, 93, 175, 275, 301, 338
    - Response: Thank you for the suggestions. Commas have been removed from Lines 45, 50, 93 (now 101), 175 (now 193), 275 (now 299), 301 (now 326), and 338 (now 365).

Additional comments from the Editor

Thank you for detailing further requirements for submission of our manuscript entitled “Assessing the Global Implications of the COVID-19 Pandemic on Cervical Cancer Screening Initiatives: A Comprehensive Evaluation”. Please see the below response to your inquiries.

5. In the online submission form, you indicated that [Insert text from online submission form here].

All PLOS journals now require all data underlying the findings described in their manuscript to be freely available to other researchers, either

1. In a public repository,

2. Within the manuscript itself, or

3. Uploaded as supplementary information.

This policy applies to all data except where public deposition would breach compliance with the protocol approved by your research ethics board. If your data cannot be made publicly available for ethical or legal reasons (e.g., public availability would compromise patient privacy), please explain your reasons by return email and your exemption request will be escalated to the editor for approval. Your exemption request will be handled independently and will not hold up the peer review process, but will need to be resolved should your manuscript be accepted for publication. One of the Editorial team will then be in touch if there are any issues."

- Response: Thank you for bringing this to our attention. All data pertinent to the question being answered in this paper are included within the manuscript itself. Additional contextual information can be obtained by contacting the corresponding author.

6. Please remove the old version of the manuscript file and retain only the revised version.
   - Response: In the re-submission, we have included a revised version of the manuscript including track changes as well as a version with the changes accepted.
7. Please send a completed 'Competing Interests' statement, including any COIs declared by your co-authors. If you have no competing interests to declare, please state "The authors have declared that no competing interests exist".
   - Response: The authors have declared that no competing interests exist.
8. Please amend your detailed Financial Disclosure statement. This is published with the article. It must therefore be completed in full sentences and contain the exact wording you wish to be published.
   c. State the initials, alongside each funding source, of each author to receive each grant. For example: ""This work was supported by the National Institutes of Health (####### to AM; ###### to CJ) and the National Science Foundation (###### to AM).""
      - Response: Please see the revised financial disclosure statement.

“This project has been funded by the United Nations Development Program - United Nations Population Fund– World Health Organization –World Bank Special Program of Research in Human Reproduction (HRP), to PA (WHO PO # 202833289). In addition, the project received support from the National Cancer Institute, National Institutes of Health, under Contract No. 75N91019D00024. The content of this publication does not necessarily reflect the views or policies of the Department of Health and Human Services, nor does mention of trade names, commercial products, or organizations imply endorsement by the U.S. Government.”

d. State what role the funders took in the study. If the funders had no role in your study, please state: “The funders had no role in study design, data collection and analysis, decision to publish, or preparation of the manuscript.”
   - Response: The funders reviewed the project proposal and provided suggestions for the interview guide. The funders also helped in the recruitment of the study participants by providing an initial introduction to the research team. Once this connection was made, the funders had no involvement in the data collection, management, and the analyses of the data. In addition, they had no role in the decision to publish or preparation of the manuscript.

Again, thank you for giving us the opportunity to strengthen our manuscript with your insightful comments and queries. We have worked diligently to incorporate your feedback and hope these revisions persuade you to accept our submission.

Sincerely,

Prajakta Adsul
