# Supplementary material for: Assessing the global implications of the COVID-19 pandemic on the cervical cancer elimination initiative
Source: PLOS Glob Public Health. 2025 Apr 28;5(4):e0004419. doi: 10.1371/journal.pgph.0004419 (PMC12036855; doi:10.1371/journal.pgph.0004419)
Supplement: Attachment — Submitted filename: PGPH-D-24-00517_reviewer.docx [file pgph.0004419.s004.docx]

PLOS Global Public Health

**Assessing the Global Implications of the COVID-19 Pandemic on Cervical Cancer Screening Initiatives: A Comprehensive Evaluation**

--Manuscript Draft--

| **Manuscript Number:** | PGPH-D-24-00517 |
| --- | --- |
| **Article Type:** | Research Article |
| **Full Title:** | Assessing the Global Implications of the COVID-19 Pandemic on Cervical Cancer Screening Initiatives: A Comprehensive Evaluation |
| **Short Title:** | Evaluating the Impact of COVID-19 on Cervical Cancer Screening Programs Across the Globe |
| **Corresponding Author:** | Prajakta Adsul, MBBS, MPH, PhD  The University of New Mexico Comprehensive Cancer Center: UNM Comprehensive Cancer Center  Albuquerque, New Mexico UNITED STATES |
| **Order of Authors:** | Anisha M Loeb, MPH |
|  | Patti Gravitt |
|  | Allison Frank |
|  | Douglas M. Puricelli Perin |
|  | Kalina Duncan |
|  | Linda Eckert |
|  | Maribel Almonte |
|  | Nathalie Broutet |
|  | Joseph Rodman |
|  | Oyeleke A Oyebamiji |
|  | Prajakta Adsul |
| **Keywords:** | Cervical cancer screening; COVID-19; Implementation Science; HPV; Health Systems Research |
| **Abstract:** | Introduction  The COVID-19 pandemic disrupted many public health programs; understanding these disruptions is critical for directing future resources. In a project studying the implementation of human papillomavirus (HPV) testing-based cervical cancer screening, we queried about the impact of the pandemic on screening programs globally.  Methods  In consultation with World Health Organization’s Regional Advisors, program managers, government officials, and clinicians involved in the implementation of HPV testing-based cervical cancer screening programs were invited to participate in semi- structured, in-depth, interviews. Interview notes and transcripts were used for inductive analysis, focusing on responses to the impact of COVID-19 pandemic on screening programs.  Results  Thirty-two interviews were conducted with participants between the age of 29 and 61 years, representing programs from 25 countries. Six key themes were noted.  Regarding disruptions, (1) the entire cancer continuum was affected, leading to delays or, in some cases even cessation of vaccination, screening, and treatment programs; and (2) a heightened sense of fear around contracting and transmitting COVID-19 |

|  | shifted government priorities and impacted healthcare delivery. Nonetheless, participants noted constructive ways in which programs leveraged the impact of the pandemic: (1) at the community level, participants were able to leverage an increased understanding and acceptance surrounding the importance of preventive health behaviors; (2) for HPV-testing: molecular laboratories became well-equipped with better technician training, increasing overall HPV-testing capacities; (3) the pandemic promoted virtual healthcare systems; and (4) for planning: shutdowns allowed time to plan for program scale-up. In many ways, the pandemic response provided evidence supporting the country’s ability to mobilize resources.  Conclusions  While disruptions were noted, the pandemic provided implementers with opportunities to strengthen screening programs, which should be further assessed in future sustainability research on cervical cancer prevention and control. |
| --- | --- |
| **Additional Information:** |  |
| **Question** | **Response** |
| **Financial Disclosure**  Enter a financial disclosure statement that describes the sources of funding for the work included in this submission and the role the funder(s) played. This includes grants and any commercial funding of the work or authors.  This statement will be typeset if the manuscript is accepted for publication.  *Please review the* [*submission guidelines*](http://journals.plos.org/globalpublichealth/s/submission-guidelines#loc-financial-disclosure-statement) *and the instructions link below for detailed requirements and guidance.* | This project has been funded in whole or in part with federal funds from the National Cancer Institute, National Institutes of Health, under Contract No. 75N91019D00024. The content of this publication does not necessarily reflect the views or policies of the Department of Health and Human Services, nor does mention of trade names, commercial products, or organizations imply endorsement by the U.S. Government. In addition, this project has been funded by the UNDP (United Nations Development Program)–UNFPA (United Nations Population Fund)–UNICEF–WHO–World Bank Special Program of Research in Human Reproduction (HRP) (contract to Dr. Adsul; WHO PO # 202833289). The funders had no role in the design of the study and analysis of data. |
| **Competing Interests**  On behalf of all authors, disclose any competing interests that could be perceived to bias this work.  This statement will be typeset if the manuscript is accepted for publication.  *Please review the instructions link below and PLOS Global Public Health’s* [*competing interests*](https://journals.plos.org/globalpublichealth/s/competing-interests) *policy to determine what information must be disclosed at* | Authors declare no competing interest. |

| *submission.* |  |
| --- | --- |
| **Data Availability**  Before publication, Authors are required to make fully available and without restriction all data underlying their findings. Please see our [PLOS Data](https://journals.plos.org/globalpublichealth/s/data-availability) [Policy](https://journals.plos.org/globalpublichealth/s/data-availability) page for detailed information on this policy.  A **Data Availability Statement**, detailing where the data can be accessed, is required at first submission. Insert your Data Availability Statement in the box below.  Please see the [data reporting](https://journals.plos.org/globalpublichealth/s/submission-guidelines#loc-data-reporting) section of our submission guidelines for instructions on what you need to include in your Data Availability Statement.  This statement will be typeset if the manuscript is accepted for publication.  *PLOS allows rare exemptions to address legal and ethical concerns. If you have legal or ethical restrictions, please use the box below to detail these in full sentences for the Journal team to consider.* | All data are provided in the manuscript and additional information may be acquired by contacting the corresponding author. |

<https://www.editorialmanager.com/pgph/download.aspx?id=310123&guid=8728c74a-b14f-4be8-a216-cec655eacf4b&scheme=1>Manuscript	[Click here to](https://www.editorialmanager.com/pgph/download.aspx?id=310123&guid=8728c74a-b14f-4be8-a216-cec655eacf4b&scheme=1)

[access/download;Manuscript;COVID_CxCaScreening_03-05-24.](https://www.editorialmanager.com/pgph/download.aspx?id=310123&guid=8728c74a-b14f-4be8-a216-cec655eacf4b&scheme=1)

**Full Title**: Assessing the Global Implications of the COVID-19 Pandemic on Cervical Cancer Screening Initiatives: A Comprehensive Evaluation

**Short Title:** Evaluating the Impact of COVID-19 on Cervical Cancer Screening Programs Across the Globe

**Authors**: Anisha M. Loeb^1^, Patti Gravitt,^2^ Allison Frank,^2^ Douglas M. Puricelli Perin,^3^ Kalina Duncan,^2^ Linda Eckert,^4,5^ Maribel Almonte, 6,7, Nathalie Broutet,^8^ Joseph Rodman^9^, Oyeleke A Oyebamiji^9^, Prajakta Adsul^9,10^

**Affiliations:**

^1^Department of Epidemiology, School of Public Health, University of Washington, Seattle, WA

^2^Center for Global Health, National Cancer Institute, Rockville, MD

3 Clinical Monitoring Research Program Directorate, Frederick National Laboratory for Cancer Research, Frederick, MD, USA

^4^Department of Obstetrics & Gynaecology, University of Washington, Seattle, WA ^5^Department of Global Health, School of Public Health, University of Washington, Seattle, WA

^6^Department of Non-Communicable Diseases, World Health Organization, Geneva, Switzerland

^7^Early Detection and Prevention Program, International Agency for Research on Cancer, Lyon, France

^8^Department of Sexual and Reproductive Health and Research, World Health Organization, Geneva, Switzerland

^9^Cancer Control and Population Sciences, University of New Mexico Comprehensive Cancer Center, Albuquerque, NM

^10^Department of Internal Medicine, University of New Mexico, Albuquerque, NM

**Funding statement:** This project has been funded in whole or in part with federal funds from the National Cancer Institute, National Institutes of Health, under Contract No. 75N91019D00024. The content of this publication does not necessarily reflect the views or policies of the Department of Health and Human Services, nor does mention of trade names, commercial products, or

organizations imply endorsement by the U.S. Government. In addition, this project has been funded by the UNDP (United Nations Development Program)–UNFPA (United Nations Population Fund)–UNICEF–WHO–World Bank Special Program of Research in Human Reproduction (HRP) (contract to Dr. Adsul; WHO PO # 202833289).

## Abstract:

**Introduction**: The COVID-19 pandemic disrupted many public health programs; understanding these disruptions is critical for directing future resources. In a project studying the implementation of human papillomavirus (HPV) testing-based cervical cancer screening, we queried about the impact of the pandemic on screening programs globally.

**Methods:** In consultation with World Health Organization’s Regional Advisors, program managers, government officials, and clinicians involved in the implementation of HPV testing- based cervical cancer screening programs were invited to participate in semi-structured, in-depth, interviews. Interview notes and transcripts were used for inductive analysis, focusing on responses to the impact of COVID-19 pandemic on screening programs.

**Results:** Thirty-two interviews were conducted with participants between the age of 29 and 61 years, representing programs from 25 countries. Six key themes were noted. Regarding disruptions, (1) the entire cancer continuum was affected, leading to delays or, in some cases even cessation of vaccination, screening, and treatment programs; and (2) a heightened sense of fear around contracting and transmitting COVID-19 shifted government priorities and impacted healthcare delivery. Nonetheless, participants noted constructive ways in which programs leveraged the impact of the pandemic: (1) at the community level, participants were able to leverage an increased understanding and acceptance surrounding the importance of preventive health behaviors; (2) for HPV-testing: molecular laboratories became well-equipped with better technician training, increasing overall HPV-testing capacities; (3) the pandemic promoted virtual healthcare systems; and (4) for planning: shutdowns allowed time to plan for program scale-up. In many ways, the pandemic response provided evidence supporting the country’s ability to mobilize resources.

**Conclusions:** While disruptions were noted, the pandemic provided implementers with opportunities to strengthen screening programs, which should be further assessed in future sustainability research on cervical cancer prevention and control.

## 1 Introduction

2 Cervical cancer (CC) continues to be commonly diagnosed and fatal among women in low- and
3 middle-income countries (LMICs) [1]. However, it is preventable through interventions such as
4 human papillomavirus (HPV) vaccination and regular CC screening followed by treatment of
5 positives. Women are vulnerable to HPV infection in the cervix across their lifespan [2], and it is
6 essential that they are screened regularly accompanied by the recommended follow-up and
7 treatment for those who are found to have screening abnormalities. In 2021, the World Health
8 Organization (WHO) put out a recommendation for the CC elimination agenda, which proposed
9 several screening and treatment algorithms, with a strong focus on using HPV DNA testing as the
10 primary screening test due to its high accuracy [3]. In response, many countries have adopted
11 and, in some cases, implemented HPV DNA screening programs following the WHO
12 recommendations; however, there is very limited information guiding the transition to and
13 implementation of population-based HPV DNA screening and treatment programs.

# 14

15 Moreover, the COVID-19 lockdown in March 2020 caused significant disruptions to health
16 systems worldwide. Due to the surge of COVID-19 cases, many health systems were unable to
17 focus on cancer screening and treatment [4]. This led to programmatic shutdowns, decreasing
18 CC screening rates by 52% globally with reductions in rates of colposcopy and invasive CC
19 diagnoses [5–11]. Public interest in cancer lessened as media coverage highlighted COVID-19
20 as the sole health threat, and Google search trends showed decreases in searches related to
21 cancer screenings [12].

# 22

23 The COVID-19 pandemic intensified barriers to seeking sexual and reproductive health care.
24 There were severe impacts to financial and human resource availability, including supply chain
25 disruptions, reduced programmatic funding, a shortage of healthcare workers, increased
26 healthcare costs, lower appointment availability, and the inability of patients to afford necessary
27 medical services [5,13–15]. The lack of resources encouraged physicians to adopt a ‘risk-based
28 approach’ in providing care, prioritizing the most vulnerable patients [14]. However, defining
29 vulnerability became difficult when physicians had to consider the severity of the disease versus
30 the number of life-years that the treatment might save. Additionally, community members lost trust
31 in the healthcare system [14,16]. This distrust coupled with fear of contracting COVID-19 and pre-
32 existing transportation barriers further limited and discouraged patients from seeking healthcare
33 services [13–15,17,18].

# 34

35 At the same time, the pandemic also highlighted potential strategies to enhance healthcare
36 delivery, particularly in relation to CC screening. The reduction of in-person clinic visits has led to
37 a greater acceptance of telemedicine visits among providers and patients, which helps to reduce
38 transportation barriers and scheduling constraints for many patients [14,15,18]. HPV testing
39 allows patients to conduct self-sampling at home, which has expanded service delivery [8,14,18].
40 Companies that conduct microbiology testing reported increases in production capacity and a rise
41 in the set-up of testing platforms to address COVID-19, which is expected to be useful for
42 expanding HPV testing post-pandemic [19]. To track COVID-19 cases, many countries opted for
43 health registries, that can be linked to national health information systems and be leveraged for
44 other diseases such as CC [8]. Lastly, the pandemic has facilitated strong collaboration and
45 coordination between health and governmental sectors, which can aid in policy and financial
46 support for preventive health programs [4,5,17,20].

# 47

48 The study is a part of a larger initiative that aimed to generate practice-based evidence for
49 strategies and approaches that support the implementation of HPV DNA-based screening and
50 treatment programs that are ongoing across the globe, guided by the Exploration, Preparation,
51 Implementation, and Sustainment Framework [21]. Since these interviews took place amidst the
52 COVID-19 pandemic, there were significant discussions around the impact of the pandemic on
53 the CC screening and treatment programs. In this paper, we characterize the impacts of COVID-
54 19 on pre-existing CC screening programs in LMICs using data from the interviews. The purpose
55 of this study is to assess strategies that were used to address the impact of COVID-19 on CC
56 screening programs in LMICs and how they could be leveraged to improve the functioning and
57 preparedness of these programs for continuity and resilience amidst future public health
58 emergencies.

# 59

## 60 Methods

61 *Recruitment*
62 All study activities were initiated after securing appropriate ethical approvals from the WHO
63 Human Reproduction Program and the University of New Mexico’s Human Research Protections
64 Office [Study ID: 21-398]. After consulting with WHO regional advisors and the study team’s
65 professional network, there were recommendations to connect with 84 practitioners/on-the-
66 ground experts involved in CC screening programs from various countries across the globe. The
67 study team, along with WHO regional advisors, sent email communications to all 84 health
68 practitioners to describe the initiative, request and confirm their interest in participation, and
69 schedule an interview. No participant compensation was offered or provided for this study.

# 70

71 *Data Collection*
72 Two study team members with implementation science and qualitative research expertise, PA
73 and PG, conducted the semi-structured interviews. The interview questions focused on the
74 effectiveness, sustainability, and scale-up potential of existing CC screening programs. Specific
75 to the analyses presented in this paper, participants were asked “How has COVID affected the
76 current implementation efforts? Were there specific changes during the pandemic that you have
77 made in program delivery? Reflecting back on the innovative strategies that you have used for
78 cervical cancer screening, are there changes you’ve made to the algorithms you used? By
79 necessity you have had to be innovative, how many of those seem like better options to continue?”
80 The Zoom virtual meeting platform was used to conduct the interviews, which lasted one to two
81 hours. Simultaneous translation was provided for non-English speakers. A supplemental
82 notetaker attended each interview to ensure interview guide concordance and assisted the
83 interviewer with relevant probes and clarifications as needed. All interviews were audio-recorded
84 and transcribed verbatim with participant consent. We achieved data saturation at 32 interviews.

# 85

86 *Data Analysis*
87 The transcripts were uploaded to Dedoose qualitative software to facilitate review, organization,
88 and analysis. The study team used a grounded theory approach to develop the COVID-19-related
89 codes and analyze the interview transcripts and notes. Grounded theory is an inductive qualitative
90 technique to identify themes that emerge from text [22]. Team members (AL, JR, and PA) trained
91 in qualitative analysis read the transcripts for each interview, and independently coded them using
92 Dedoose. During initial coding, the study team met regularly to compare codes and resolve
93 discrepancies, by the third coder. Team discussions led to the identification of six major themes
94 from the data, and the team generated summary statements and narratives based on these
95 themes. Collectively, these themes represent the impact of COVID-19 on the CC screening and
96 treatment programs and were used to organize the results. Participant responses were de-
97 identified to only include the WHO region and profession. Quotes were selected to represent each
98 theme.

# 99

## 100 Results

101 *Participants*
102 The study team completed 32 interviews between June and October 2022. Participants were aged
103 between 29-61 years and came from 25 countries including: 10 from the Americas region (AMR),
104 10 from the Africa region (AFR), four from the Europe region (EUR), one from the Eastern
105 Mediterranean region (EMR), three from the Western Pacific region (WPR), and five from the
106 South-East Asia region (SEAR). Participant professions included health ministry officials,
107 clinicians, program managers, clinicians, and epidemiologists.

# 108

109 *Theme 1: The COVID-19 pandemic had an impact on healthcare facilities and human resources*
110 *across the CC continuum*
111 Almost all participants noted that the lockdown measures implemented to combat COVID-19
112 caused the shutdown of healthcare facilities and public health programs, resulting in a negative
113 impact on preventive services. The rates of HPV vaccination and CC screening rates notably
114 decreased. Participants reported full-scale programmatic shutdowns or delays in pilot programs,
115 setting back progress made towards CC elimination. Interestingly, there were some countries
116 where programs continued uninterrupted by COVID-19. However, participants noted that health
117 systems overall were unprepared for the pandemic. A primary-level health professional from the
118 AMR region noted:

# 119

120 *“Because our health system …was improper. I think that, that the worse thing was when*
121 *we had to stop all the programs, all the prevention programs were stopped and … we*
122 *took up a bad idea to shut down all the primary care facilities. All, all were shut down at*
123 *the beginning of COVID-19. So that was crazy because all, all the people were sick at*
124 *the hospitals and we don't have enough hospitals, but that decision was made and all*
125 *the programs …that weren't COVID-19 were shut down”*

# 126

127 All participants noted that to address the global emergency, financial and human resources were
128 redirected from health programs, negatively impacting the procurement of HPV DNA tests, the
129 availability of trained professionals, and overall testing capacity. This resulted in a backlog in
130 diagnostic testing, which led to a subsequent backlog in treatment referrals. A lead physician in
131 the AFR region noted:

# 132

133 *“Outpatient was literally closed, and they turned that area into a COVID-19 area. That*
134 *meant that the screening availability was also quite reduced. Then there was a time the*
135 *referrals were stopped.”*

# 136

137 Many healthcare workers were mobilized toward COVID-19-related needs, and some were
138 diverted to providing primary care to patients living with the virus. The diversion of these trained
139 professionals impacted the scale and quality of care during the pandemic, but also continued to
140 impact public health programs with the need for refresher trainings. A tertiary-level health
141 professional from the SEAR region noted the impacts of COVID-19 on the workforce for VIA:

# 142

143 *“So, the main challenges retaining the trained staff in the same program when COVID-*
144 *19 pandemic stuck. All the people trained for VIA [visual inspection using acetic acid],*
145 *they are lost. They have been scattered into different health programs, so now getting*
146 *them back… to the same program by itself is a challenge.”*

# 147

148 Participants noted that the pandemic resulted in a clear need to collect cervical samples with a
149 limited number of healthcare workers, leading to favoring of HPV DNA tests which allowed self-
150 sampling. Many noted that self-sampling could increase screening rates by reaching more women
151 and many interviewees indicated patient preference towards self-sampling increasing their
152 comfort and eliminating transportation barriers. A program director in the AFR region noted:

# 153

154 *“Due to COVID-19, we were, I don’t want to say forced, but were kind of forced to think*
155 *through a more innovative way of getting samples without having women come to the*
156 *facility. This is where we added the self-sampling arm both for the women who are in the*
157 *facility. They will be given the opportunity to self-sample within the facility. I agree, for*
158 *me, this is the most cost-effective way of self-sampling because it eliminates first the*
159 *stigma around a pelvic exam, but it also eliminates the requirement for a speculum*
160 *which is a challenge in our setting, procuring as many speculums as needed.”*

# 161

162 *Theme 2: The fear of contracting and transmitting COVID-19 shifted government priorities and*
163 *impacted healthcare delivery*
164 Several participants pointed out that the success and sustainability of programs depend on
165 governmental or ministry support. During the pandemic, participants stressed that any focus
166 governmental officials had on implementing CC screening programs, as well as human resources,
167 were diverted to COVID-19 testing and care, which significantly impeded the already poorly-
168 funded programs. A public health specialist in the AFR region noted:

# 169

170 *“COVID-19 vaccine campaigns disrupted service delivery at health care facilities and*
171 *decreased government spending for cervical cancer related services due to competing*
172 *priorities.”*

# 173

174 This disruption not only impacted preventive measures during the pandemic as reported by the
175 interviewees, but when countries were able to prioritize CC, it was difficult to resume
176 programmatic implementation. Thus, many existing programs were affected for longer than
177 initially thought, and screening and vaccination rates remained consistently low.

# 178

179 Several participants expressed widespread fear about COVID-19 among individuals seeking
180 healthcare as well as frontline workers. A Ministry of Health representative from the AMR region
181 noted:

# 182

183 *“So, I think the human resources definitely got hit hard where we had to sacrifice primary*
184 *care interventions. Fear from COVID-19 was present on the demand side and the supply*
185 *side. We rushed people away from the health care facilities so that they don’t get*
186 *infected, or they don’t infect us.”*

# 187

188 It was reported that this fear also led to physical distancing protocols, which negatively impacted
189 the delivery of in-person healthcare services as well as community engagement and outreach.
190 Screening rates dropped in populations who especially rely on community campaigns for
191 education and service delivery. A lead physician from the AFR region summarized:

# 192

193 *“We had this recommendation that come out for social distancing purposes, you can no*
194 *longer conduct outreach, especially during the peaks. That actually resulted in reduced*
195 *screening rates.”*

# 196

197 *Theme 3: Awareness around the COVID-19 pandemic led to an increase in understanding about*
198 *preventive health services*

# 199

200 Almost all participants acknowledged that the COVID-19 pandemic increased public awareness
201 of viruses and how they are transmitted, and that such awareness led to a greater acceptance of
202 preventive health measures such as vaccinations and early detection. A professor from the AMR
203 region highlighted the benefits of this increased awareness and emphasized the need for further
204 messaging to promote screening programs:

# 205

206 *“I think one of the critical things, I believe that …everybody by the way after COVID-19,*
207 *understands what a virus is, what a virus can do, and what a molecular test is. That's a*
208 *big advantage. They understand that somebody can stay with the virus. They even are*
209 *asking me if they can be vaccinated because they also think about COVID-19.*
210 *Unfortunately, we have to tell them that we can vaccinate only the girls for the future, but*
211 *they already had the virus, so at this point what we need to do is to find that the virus is*
212 *there, and we have to treat them. We’re using that message, so communication is also*
213 *an important thing.”*

# 214

215 As noted above, this perceived improvement in awareness among the populations was an
216 important advantage for public health communications for CC prevention.

# 217

218	*Theme 4: Preparedness for COVID-19 testing had an impact on CC screening testing capacities*

# 219

220 According to several participants, the laboratories used the same testing platforms for various
221 diseases such as HPV, HIV, and TB. PCR tests for COVID-19 also leveraged these same testing
222 platforms and thus countries were able to use pre-existing testing platforms to address the
223 pandemic. This meant that the testing capacity for CC was undercut, which led to delays in testing
224 and increased chances of loss to follow-up. A health authority professional from the AFR region
225 noted:

# 226

227 *“Oh, COVID-19 testing. Another thing is COVID-19 testing all platforms. Oh my God.*
228 *That was a nightmare because all platforms were busy with COVID-19 testing. So, all*
229 *the HPV testing were put on hold for like--in some places, even for two months.”*

# 230

231 However, to combat the scale of the pandemic, more individuals received training and became
232 familiar with molecular testing platforms. Additional financial support and improvement of physical
233 infrastructure increased the testing capacities in these laboratories. These advancements in
234 testing capabilities can be leveraged for HPV testing when the incidence of COVID-19 slows
235 down. A health authority professional from the AMR region noted, candidly:

# 236

237 *“But, with the change of COVID-19, biomolecular testing is no longer rocket science. So*
238 *there’s an opportunity to think of other technologies that could be used, and rethink,…*
239 *what if now we could do this dry swabs... [if] we need a PCR machine and we could do*
240 *this in a regional hospital...So I think there’s an opportunity to use technologies that are*
241 *not going to be used, hopefully, for SARs, COVID 2 in the future, that are in a lot of*
242 *these hospitals that had no biomolecular testing in the past. So, that’s another*
243 *opportunity.”*

# 244

245 Participants shared many lessons learned from implementing widespread COVID-19 testing that
246 could inform future processes for HPV testing. This includes the use of barcodes to ensure quality,
247 text messaging to communicate results linked to health information systems, and the
248 centralization of services, as explained by an epidemiologist in the EUR region:

# 249

250 *“We take one positive advantage that year because that year was many connections*
251 *with Directory of Public Health of the District with the Institute of Public Health. Because*
252 *they collected the sample for COVID-19 and they sent all these samples in the lab of the*
253 *Institute. Because the lab of the Institute of [country name removed] was the main lab for*
254 *analyzing COVID-19 tests. There were many connections, so this is the positive to cover*
255 *all the program in a few months, the program of the screening.”*

# 256

257 *Theme 5: The COVID-19 pandemic revealed fragmentation of healthcare systems and also*
258 *promoted a move towards virtual healthcare systems*
259 Some participants shared concerns about the impact of the pandemic on the fragmented
260 healthcare systems, which are crucial for screening programs to remain successful. An
261 epidemiologist from the AMR region highlighted this problem, describing through a scenario of
262 managing patients living with COVID-19 compared to other primary care services:

# 263

264 *“Okay, if a woman is HPV positive at the center, where will they be sent? To hospital A.*
265 *Okay, these women were sent to the hospital for that’s where they’ll get a*
266 *colposcopy…when hospital A is closed right now. Oh, wait, hospital A is close? Yeah,*
267 *because of COVID-19. You only take COVID-19 patients? Yes. Where are you going to*
268 *send the women? To hospital A. But you’re saying it’s closed. Yes. They’re going to be*
269 *brought to the hospital. They’re going to return back. Yes. And then, what? They will*
270 *have to figure something else out.”*

# 271

272 While there were challenges noted in healthcare delivery, many participants noted that community
273 health education programs and professional training programs could be moved to an online
274 delivery mode. Some participants even mentioned they preferred the virtual modality and planned
275 to continue using it after the pandemic, due to its reach. Several participants noted that physicians
276 were more accepting of telemedicine appointments, which increased capacity under social-
277 distancing protocols and will continue to do so if patients prefer virtual appointments. A tertiary-
278 level health professional from the WPR region noted:

# 279

280 *“Yeah. And, especially after COVID-19 because during COVID-19, we carried out many*
281 *activities only through the live-stream. I mean, even for health counselling are through*
282 *apps, or through live-streaming on You-Tube, etc. Now, they are more-or-less familiar*
283 *with that kind of tele-scheduling.”*

# 284

285 *Theme 6: Additional downtime due to the pandemic was advantageous in the future of*
286 *programmatic implementation and CC screening potentials*

# 287

288 Some participants mentioned that the shutdowns and downtime in implementation efforts allowed
289 them to pivot and at times modify their efforts on targeting specific high-risk groups and planning
290 community campaigns. A lead physician in the AFR region cited:

# 291

292 *“But what we then did, is we utilized that sort of downtime to plan for sort of rapid scale*
293 *up post pandemic. Yeah. What we did is to actually try and mobilize resources, find out*
294 *what is needed, the gloves, do you need to procure more speculums and things like that.*
295 *Then to also communicate to the respective cadres within the regions that we plan to*
296 *have a campaign, maybe after three months or so. We had these targets that we*
297 *couldn't get. When the COVID-19 restrictions are relaxed, we are going to have a*
298 *massive campaign, and we need to have massive tracking of patients.”*

# 299

300 Moreover, the manner in which countries around the world united and mobilized resources to
301 address COVID-19 demonstrated to the participants, the hidden potential to address other global
302 health disparities. Participants reported feeling more confident in improving health outcomes for
303 CC. A tertiary-level health professional from the SEAR region elaborated:

# 304

305 *“Again, the COVID-19 testing only like gives us confidence. How they centralized. When*
306 *the government was able to do that COVID-19 testing in such a huge manner in a very*
307 *short time with limited resources. So, it should be possible for HPV DNA also, so that*
308 *gives us the confidence.”*

# 309

## 310 Discussion

311 The emerging healthcare delivery literature has made it abundantly clear that COVID-19 had a
312 substantial impact on health systems across the globe. On-the-ground experts included in this
313 study perceived a shift in government priorities, which resulted in shifts in human and financial
314 resources. Public health initiatives aimed at reducing COVID-19 transmission as well as
315 community fear surrounding an unknown virus effectively decreased the opportunities that
316 individuals had to go into clinics in-person to receive CC preventive services, including vaccination
317 and screening. This effect was reported in a number of studies, who quantified vaccination,
318 screening, and treatment rates, comparing pre-pandemic to mid-pandemic rates [6–10].
319 Nonetheless, the pandemic presented global healthcare systems with opportunities to find
320 strategies to strengthen preventive services and programs. Community members gained
321 familiarity in modes of viral transmission and ways to protect oneself, which can be used to
322 promote CC preventive services. The laboratories were equipped with PCR machines for COVID-
323 19 testing and an increasing number of technicians were trained to run these tests. Because these
324 machines are not specific to COVID-19, there exists increased infrastructure for other molecular
325 tests such as HPV testing. Molecular diagnostic companies are also optimistic in the rise of
326 molecular testing potentials, as cited in Poljak et. al [19].

# 327

328 While many health systems revealed poor coordination efforts, there was a need to shift the ways
329 in which some practices were conducted. The rise in virtual activities associated with social
330 distancing protocols resulted in increased acceptance and usage of telemedicine and web-
331 delivered trainings for the healthcare professional workforce. HPV DNA tests, in which samples
332 can be collected without the need of a clinician, gained increased acceptance among healthcare
333 professionals and public health decision-makers as a method to continue screening in times of
334 health emergencies, despite the cost. Numerous studies highlighted the rise of telemedicine and
335 self-sampling as ways that CC preventive efforts continued to function and can increase rates
336 even post-pandemic [8,14,15,18]. The additional time experts had during programmatic holds or
337 shutdowns allowed for increased planning efforts and strategies to target those most at risk.
338 Ultimately, the way the global community mobilized together to tackle COVID-19, proved the
339 ability for health systems to work together to help prevent disease transmission and outbreaks.

# 340

341 Some prior studies mentioned the lack of impact of COVID-19, especially on colposcopy and
342 treatment rates [7–9]. While we did not directly find these results, we did become aware of the
343 differential impact COVID-19 had on regions around the world. For instance, many African
344 countries noted short-term shutdowns of health services. Moreover, studies on high-income
345 countries expressed the inability of patients to afford healthcare services during the pandemic
346 [14]. As our study focused primarily on LMICs, whose populations experience these hardships
347 under non-pandemic conditions, we did not find increased inability to afford healthcare be a
348 driving consequence of COVID-19.

# 349

350 This study had some limitations, which need to be acknowledged. The study sample was
351 comprised of public health and health care practitioners who responded to email invitations
352 among those selected by WHO regional advisors and study team members based on their
353 expertise, knowledge, and involvement in CC screening programs in their countries. While the
354 initial goal was to obtain representation from each WHO region, some were less responsive than
355 others that maybe reflective of overburdened healthcare systems during the pandemic. Thus,
356 study conclusions may not reflect experiences from individuals in countries that were not a part
357 of the study. The data presented were validated through notes taken during interviews and
358 transcripts developed from audio recordings. However, we were unable to conduct participant
359 checking to ensure our understanding of certain points was as the interviewee intended. Lastly,
360 as the pandemic was still a global emergency impacting health systems during the time we
361 conducted the interviews, some responses were theoretical as to how certain systems may
362 change because of COVID-19, and future research could generate the concrete evidence to
363 confirm these hypotheses. Further, the primary focus of our study was on screening services so
364 when participants did comment on vaccination, precancer treatment, and cancer treatment rates,
365 we did not probe to learn more about the impact of COVID-19 on those rates.

# 366

367 The study has many strengths. To the best of our knowledge, this was one of the first studies to
368 gather global in-depth data, using a qualitative method of inquiry, in capturing the practice-based
369 understanding for the implementation of CC screen and treatment programs. We reached a point
370 of data saturation, which means that interviewers and analysts noted repetitions of concepts and
371 ideas presented by the participants. This allowed us to feel confident in drawing inferences about
372 the impact of the COVID-19 pandemic from these data. The semi-structured nature of the in-depth
373 interviews allowed us to strengthen the interview guide as more interviews were conducted. We
374 interviewed individuals involved in many aspects of CC screening programs, from the research
375 up to the national policy-making level, which allowed us to gain multiple insights and perspectives
376 on the impacts of COVID-19. Our future studies, post-pandemic, will evaluate whether these
377 strategies have been kept in place and whether they have helped to sustain and strengthen CC
378 screening programs across the globe.

# 379

380 Overall, study findings suggest that strategies that helped keep health services functional
381 throughout the pandemic have the potential to be leveraged to improve CC prevention for rapid
382 progress towards CC elimination.

# 383

## 384 Acknowledgments

385 We sincerely thank the study participants for their insights and time.

## 386 References

387 1. Singh D, Vignat J, Lorenzoni V, Eslahi M, Ginsburg O, Lauby-Secretan B, et al. Global
388 estimates of incidence and mortality of cervical cancer in 2020: a baseline analysis of the
389 WHO Global Cervical Cancer Elimination Initiative. The Lancet Global Health. 2023 Feb
390 1;11(2):e197–206.
391 2. Guo F, Hirth JM, Berenson AB. Comparison of HPV prevalence between HPV-vaccinated
392 and non-vaccinated young adult women (20–26 years). Hum Vaccin Immunother. 2015
393 Sep 16;11(10):2337–44.
394 3. WHO guideline for screening and treatment of cervical pre-cancer lesions for cervical
395 cancer prevention: Use of mRNA tests for human papillomavirus (HPV). In: 2nd ed.
396 Geneva: World Health Organization; 2021 [cited 2023 Dec 7]. Available from:
397 https://[www.ncbi.nlm.nih.gov/books/NBK576617/](http://www.ncbi.nlm.nih.gov/books/NBK576617/)
398 4. Huguet N, Danna M, Baron A, Hall J, Hodes T, O’Malley J, et al. Delivery of Cervical and
399 Colorectal Cancer Screenings During the Pandemic in Community Health Centers. Med
400 Care. 2023 Aug;61(8):554–61.
401 5. Puricelli Perin DM, Elfström KM, Bulliard JL, Burón A, Campbell C, Flugelman AA, et al.
402 Early assessment of the first wave of the COVID-19 pandemic on cancer screening
403 services: The International Cancer Screening Network COVID-19 survey. Prev Med. 2021
404 Oct;151:106642.
405 6. Desta AA, Woldearegay TW, Gebremeskel E, Alemayehu M, Getachew T, Gebregzabiher
406 G, et al. Impacts of COVID-19 on essential health services in Tigray, Northern Ethiopia: A
407 pre-post study. PLOS ONE. 2021 Aug 27;16(8):e0256330.
408 7. Martins TR, Witkin SS, Mendes-Corrêa MC, de Godoy AS, Cury L, Balancin ML, et al.
409 Impact of the COVID-19 Pandemic on Cervical Cancer Screening in São Paulo State,
410 Brazil. Acta Cytol. 2023 Jan 20;1–7.
411 8. Cruz-Valdez A, Palacio-Mejía LS, Quezada-Sánchez AD, Hernández-Ávila JE, Galicia-
412 Carmona T, Cetina-Pérez L del C, et al. Cervical cancer prevention program in Mexico
413 disrupted due to COVID-19 pandemic: Challenges and opportunities. Frontiers in Oncology
414 [Internet]. 2023 [cited 2023 Jul 27];13. Available from:
415 https://[www.frontiersin.org/articles/10.3389/fonc.2023.1008560](http://www.frontiersin.org/articles/10.3389/fonc.2023.1008560)
416 9. Lucas E, Murillo R, Arrossi S, Bárcena M, Chami Y, Nessa A, et al. Quantification of impact
417 of COVID-19 pandemic on cancer screening programmes – a case study from Argentina,
418 Bangladesh, Colombia, Morocco, Sri Lanka, and Thailand. eLife. 12:e86527.
419 10. Ferrara P, Dallagiacoma G, Alberti F, Gentile L, Bertuccio P, Odone A. Prevention,
420 diagnosis and treatment of cervical cancer: A systematic review of the impact of COVID-19
421 on patient care. Prev Med. 2022 Nov;164:107264.
422 11. Lee J, Ismail-Pratt I, Machalek DA, Kumarasamy S, Garland SM. The recovery strategies to
423 support cervical cancer elimination in lower-and middle-income countries (LMICs) following
424 COVID-19 disruptions. Preventive Medicine Reports. 2023 Oct 1;35:102291.
425 12. Cohen SA, Ebrahimian S, Cohen LE, Tijerina JD. Online public interest in common
426 malignancies and cancer screening during the COVID-19 pandemic in the United States. J
427 Clin Transl Res. 2021 Dec 28;7(6):723–32.
428 13. Murewanhema G. Reduced cervical cancer screening in Zimbabwe as an indirect impact of
429 the COVID-19 pandemic: implications for prevention. Pan Afr Med J. 2021 Feb 5;38:131.
430 14. Wentzensen N, Clarke MA, Perkins RB. Impact of COVID-19 on cervical cancer screening:
431 Challenges and opportunities to improving resilience and reduce disparities. Prev Med.
432 2021 Oct;151:106596.
433 15. Ng’andu M, Mesic A, Pry J, Mwamba C, Roff F, Chipungu J, et al. Sexual and reproductive
434 health services during outbreaks, epidemics, and pandemics in sub-Saharan Africa: a
435 literature scoping review. Systematic Reviews. 2022 Aug 9;11(1):161.
436 16. DeBoer RJ, Fadelu TA, Shulman LN, Van Loon K. Applying Lessons Learned From Low-
437 Resource Settings to Prioritize Cancer Care in a Pandemic. JAMA Oncology. 2020 Sep
438 1;6(9):1429–33.
439 17. Alfaro K, Soler M, Maza M, Flores M, López L, Rauda JC, et al. Cervical Cancer Prevention
440 in El Salvador: Gains to Date and Challenges for the Future. Cancers (Basel). 2022 Jun
441 3;14(11):2776.
442 18. Rodriguez NM, Brennan LP, Claure L, Balian LN, Champion VL, Forman MR. Leveraging
443 COVID-era innovation for cervical cancer screening: Clinician awareness and attitudes
444 toward self-sampling and rapid testing for HPV detection. PLoS One.
445 2023;18(3):e0282853.
446 19. Poljak M, Cuschieri K, Waheed DEN, Baay M, Vorsters A. Impact of the COVID-19
447 pandemic on human papillomavirus-based testing services to support cervical cancer
448 screening. Acta Dermatovenerol Alp Pannonica Adriat. 2021 Mar;30(1):21–6.
449 20. Puricelli Perin DM, Christensen T, Burón A, Haas JS, Kamineni A, Pashayan N, et al.
450 Interruption of cancer screening services due to COVID-19 pandemic: lessons from
451 previous disasters. Prev Med Rep. 2021 May 17;23:101399.
452 21. Aarons GA, Hurlburt M, Horwitz SM. Advancing a Conceptual Model of Evidence-Based
453 Practice Implementation in Public Service Sectors. Adm Policy Ment Health. 2011 Jan
454 1;38(1):4–23.
455 22. Glaser B, Strauss A. The discovery of grounded theory: strategies for qualitative research.
456 5th ed. New Brunswick: Aldine Transaction; 2010. 271 p.

457
